# A systematic review of moral reasons on orphan drug reimbursement

**DOI:** 10.1186/s13023-021-01925-y

**Published:** 2021-06-30

**Authors:** Bettina M. Zimmermann, Johanna Eichinger, Matthias R. Baumgartner

**Affiliations:** 1 Institute for Biomedical Ethics, University of Basel, Bernoullistrasse 28, 4056 Basel, Switzerland; 2 Institute for History and Ethics in Medicine, Technical University of Munich School of Medicine, Technical University of Munich, Munich, Germany; 3 Division of Metabolism and Children’s Research Center, University Children’s Hospital Zurich, University of Zurich, Zurich, Switzerland

**Keywords:** Rare diseases, Orphan diseases, Orphan drugs, Reimbursement, Ethics, Allocation of resources, Publicly funded healthcare systems, Systematic review of reasons

## Abstract

**Background:**

The number of market approvals of orphan medicinal products (OMPs) has been increasing steadily in the last 3 decades. While OMPs can offer a unique chance for patients suffering from rare diseases, they are usually very expensive. The growing number of approved OMPs increases their budget impact despite their low prevalence, making it pressing to find solutions to ethical challenges on how to fairly allocate scarce healthcare resources under this context. One potential solution could be to grant OMPs special status when considering them for reimbursement, meaning that they are subject to different, and less stringent criteria than other drugs. This study aims to provide a systematic analysis of moral reasons for and against such a special status for the reimbursement of OMPs in publicly funded healthcare systems from a multidisciplinary perspective.

**Results:**

With a systematic review of reasons, we identified 39 reasons represented in 243 articles (scientific and grey literature) for and against special status for the reimbursement of OMPs, then categorized them into nine topics. Taking a multidisciplinary perspective, we found that most articles came from health policy (n = 103) and health economics (n = 49). More articles took the position for a special status of OMPs (n = 97) than those against it (n = 31) and there was a larger number of reasons identified in favour (29 reasons) than against (10 reasons) this special status.

**Conclusion:**

Results suggest that OMP reimbursement issues should be assessed and analysed from a multidisciplinary perspective. Despite the higher occurrence of reasons and articles in favour of a special status, there is no clear-cut solution for this ethical challenge. The binary perspective of whether or not OMPs should be granted special status oversimplifies the issue: both OMPs and rare diseases are too heterogeneous in their characteristics for such a binary perspective. Thus, the scientific debate should focus less on the question of disease prevalence but rather on how the important variability of different OMPs concerning e.g. target population, cost-effectiveness, level of evidence or mechanism of action could be meaningfully addressed and implemented in Health Technology Assessments.

**Supplementary Information:**

The online version contains supplementary material available at 10.1186/s13023-021-01925-y.

## Background

Orphan medicinal products (OMPs) are highly specialized treatments for very small groups of patients. The definition of OMPs slightly varies between regulations. According to the European Commission Regulation [1], an orphan designation pertains exclusively to OMPs that are “intended for the diagnosis, prevention or treatment of a life-threatening or chronically debilitating condition affecting not more than five in 10,000 persons in the Community” (Article 2.1) or that fulfill the condition that “without incentives it is unlikely that the marketing of the medicinal product in the Community would generate sufficient return to justify the nece[s]sary investment” (Article 2.2). The sponsor of an orphan drug designation must additionally establish that there exist no satisfactory alternatives or that the new drug is better than the existing alternatives (Article 2.3) [1, 2].

Because developing drugs for rare diseases had not been lucrative for the pharmaceutical industry, the United States started incentivizing research and development of OMPs with the Orphan Drug Act in 1983 [3]. The European Union has followed this attempt with the Orphan Regulation in 1999 (Reg. no 141/2000) [4]. The incentives vary between jurisdictions but often include market exclusivity, tax exemptions, research funding, and free-of-charge research advice. Moreover, OMP incentives may be connected to more general incentive programs, such as accelerated market authorization procedures, the option for off-label use (meaning the use of a drug before its official approval for a specific therapy), and compassionate use programs [5]. Since the implementation of these incentive programs, the number of approved OMPs has been growing exponentially [6]. However, not all approved OMPs are targeted towards rare diseases: In light of the ongoing research progress in precision medicine, targeted therapies for common diseases might also fall into the definition of orphan drug regulations. Still, a US-based empirical study did not find targeted therapies to mainly cause the increase in OMP approvals [7].

Drugs are approved for marketing and orphan designation by medical agencies (such as the Federal Drug Administration in the United States and the European Medical Agency in the European Union) based on their efficacy and safety. In publicly funded healthcare systems, however, a separate country-specific process decides whether or not an approved drug is reimbursed. This process may include a Health Technology Assessment (HTA), which assesses the value of medical products as supported by clinical evidence and cost-effectiveness analyses.

OMPs share the characteristic of aiming to treat small populations with targeted therapies, which leads to higher costs and difficulties in obtaining clinical evidence [8]. As prices are often high and negatively correlate with disease prevalence [9], OMPs are often not cost-effective [10]. Recently approved curative OMPs have well exceeded previous pricing standards: onasemnogene abeparvovec, for instance, a gene replacement therapy attempting to cure Spinal Muscular Atrophy with a one-time treatment, costs more than 2 million US$. Still, the drug has been considered cost-effective with this price tag compared to alternatives if administered early [11] and has been approved for reimbursement in various countries [e.g., 12–14]. Consequently, many OMPs are reimbursed regardless of their high prices [e.g., 15, 16], thereby being granted special status. Special status, in this context, is defined as the application of differential criteria in reimbursement decision-making for OMPs compared to non-OMPs.

Because of the growing number of expensive OMPs, their reimbursement through public health insurances is increasingly manifesting itself as a moral dilemma for decision-makers: Basing OMP reimbursement on rules of exception is becoming unsustainable, as financing many expensive OMPs within a publicly funded healthcare system inevitably leads to cuts in other healthcare areas [17]. Not reimbursing any orphan drugs, however, is equally problematic given the immense need of patients who depend on them.

Theories of distributive justice provide some guidance in the ethical challenge of healthcare resource allocation [18, 19]. First, utilitarianism intends to maximise overall population-wide well-being [20] and is traditionally a guiding principle for health policy; the element of cost-effectiveness in the traditional reimbursement decision criteria is based on this utilitarian principle. Second, egalitarians represent the cause that everyone is treated equally, but some theorists acknowledge that certain compensation might be required for the disadvantaged to achieve equality [21]. Third, the libertarian position stresses individual freedom and calls for minimizing state interventions. Therefore, supporting the disadvantaged is only recognized when based on voluntary actions of individuals [22]. Fourth, communitarians contrast from libertarian positions with their emphasis on community interests. They call for policies that emphasize the needs and interests of communities over the interests and needs of individuals [23]. Finally, the “rule of rescue”, albeit it is not an established theory of distributive justice but rather a practice-oriented principle, calls for saving identifiable individuals no matter the cost if they are dying soon and have the chance to be rescued. It is thought to be a rule of exception and has been implemented in the Australian OMP reimbursement policy [24–29]. Moreover, because OMP reimbursement points to an ethical challenge without any clear-cut solution, many authors call for public debates to discuss its challenges and subsequent strategies that are legitimated via deliberative democratic processes [30, 31].

In light of the increasing number of high-priced OMP approvals and the unsolved ethical challenges concerning their reimbursement in publicly funded healthcare systems, this study aims to provide a systematic analysis of moral reasons for and against special status for the reimbursement of OMPs in publicly funded healthcare systems from an interdisciplinary perspective. To our knowledge, there is not yet a systematic review of the ethical considerations regarding OMP reimbursement available to date. The following research questions are addressed: RQ1—What reasons in favour or against the special status for reimbursing OMPs are discussed in the scientific and grey literature? RQ2—What reasons are dominating the scientific discourse? RQ3—What disciplines contribute to this debate and how heterogeneous is the field? RQ4—To what countries and regions worldwide does this debate refer to?

## Methods

A systematic review of reasons [32] was the methodological basis for this study. This method allows for a systematic assessment of the moral reasons for and against special status for the reimbursement of OMPs as compared to non-orphan drugs in reimbursement decision-making. It aims to synthesize them rather than to assess their adequacy and quality [33]. Moral reasons were defined as arguments about what decision, from a moral perspective, ought to be the right one [34].

### Search strategy

Scientific databases were systematically searched for relevant articles. They were selected to cover a variety of research fields relevant to the topic: new PubMed (medicine), EMBASE via Elsevier (biomedicine), CINAHL (nursing), Web of Science Core Collection (interdisciplinary), Philosopher’s Index with full text via EBSCOhost (philosophy), HeinOnline (law), Open Grey (grey literature), and Google Search was targeted for stakeholder position papers (grey literature). Additional file 1 provides detailed documentation of the search strategy, including database-specific search strings. No time restrictions were set for this study.

### Article selection

Article duplicates were removed using the identifying function in Citavi 6.0, checking each duplicate manually before removal. BZ screened the articles' titles and abstracts and excluded articles that were not about reimbursement issues concerning rare diseases or OMPs. The remaining articles were assessed based on the full texts and were included if (1) there was at least one moral reason for or against special status for the reimbursement of OMPs; (2) the format was either a book chapter, research article, commentary, PhD thesis, editorial, conference paper (abstract collections were excluded), position paper or blog written by specialists and targeted to a specialised audience (articles targeted to a general audience, such as newspaper articles, were excluded); (3) full texts were in English or German language; (4) full texts were available. In Additional file 2, we report how many articles were excluded for what reasons. The first 556 (55%) full texts were assessed by two researchers (BZ and JE) independently and compared, discrepancies were discussed and inclusion decisions made based on agreement. The remaining full texts were assessed by BZ alone, but those that were difficult to judge were double-checked by JE. References of all articles were screened for additional articles, but reference screening was limited to the references that were cited in relevant text passages.

The analysis was limited to articles discussing OMP for rare diseases, thereby excluding those relevant to neglected diseases (diseases that are predominantly present in developing countries), distribution issues related to developing countries (unless they concern drugs with an orphan designation), issues related to pediatric drugs (unless they concerned drugs with an orphan designation) and articles only covering reimbursement for personalized treatment or other groups of treatment that might or might not fall under the orphan indication. We also did not consider articles that discussed pricing issues without considering reimbursement.

### Data extraction and analysis

To address RQ1 and RQ2, we applied an inductive procedure, where we systematically identified and extracted moral reasons for or against special status for the reimbursement of OMPs; then we sorted and categorised them in several iterative steps. First, two researchers (BZ and JE) separately identified text passages that included a moral reason from three deliberately selected articles with a particular focus on reasons for and against special status for the reimbursement of OMPs. Text passages were compared and their discrepancies discussed. Every text passage identified was assigned a “narrow reason”, a code that summarized its core argument. If multiple arguments were combined in one text passage, multiple codes were subsequently applied to the same text passage. In two additional rounds, the same procedure was applied to another 42 randomly selected articles. Subsequently, these identified “narrow reasons” were preliminarily categorized and discussed based on the selected 45 articles. The remaining data extraction was done by BZ alone, following and refining this preliminary categorical system.

To address the heterogeneity of disciplines discussing moral reasons (RQ3) and the geographical distribution (RQ4), variables were extracted deductively using a predefined codebook (see Additional file 3) that was inspired by previous methodological considerations for systematic reviews of reasons [32]. Disciplines were assessed based on the content of the articles; if the content was not unambiguously assignable to one discipline, the first author's affiliation was considered; if it was a multidisciplinary affiliation the field of the journal was additionally considered. Geographical regions were assessed by first collecting, for each article, to what country or countries the article was referring to. From this extracted information, an exhaustive list of categories was built and applied to each article (see Additional file 3).

For data analysis, all reasons mentioned above were counted, including those citing reasons from other authors, because we were interested in the relative prevalence of the reasons. Data collection and analysis were supported by IBM SPSS Statistics 25 (for graphical representation and descriptive statistics) and MaxQDA 2020 (for coding and categorizing reasons).

## Findings

**Fig. 1:**
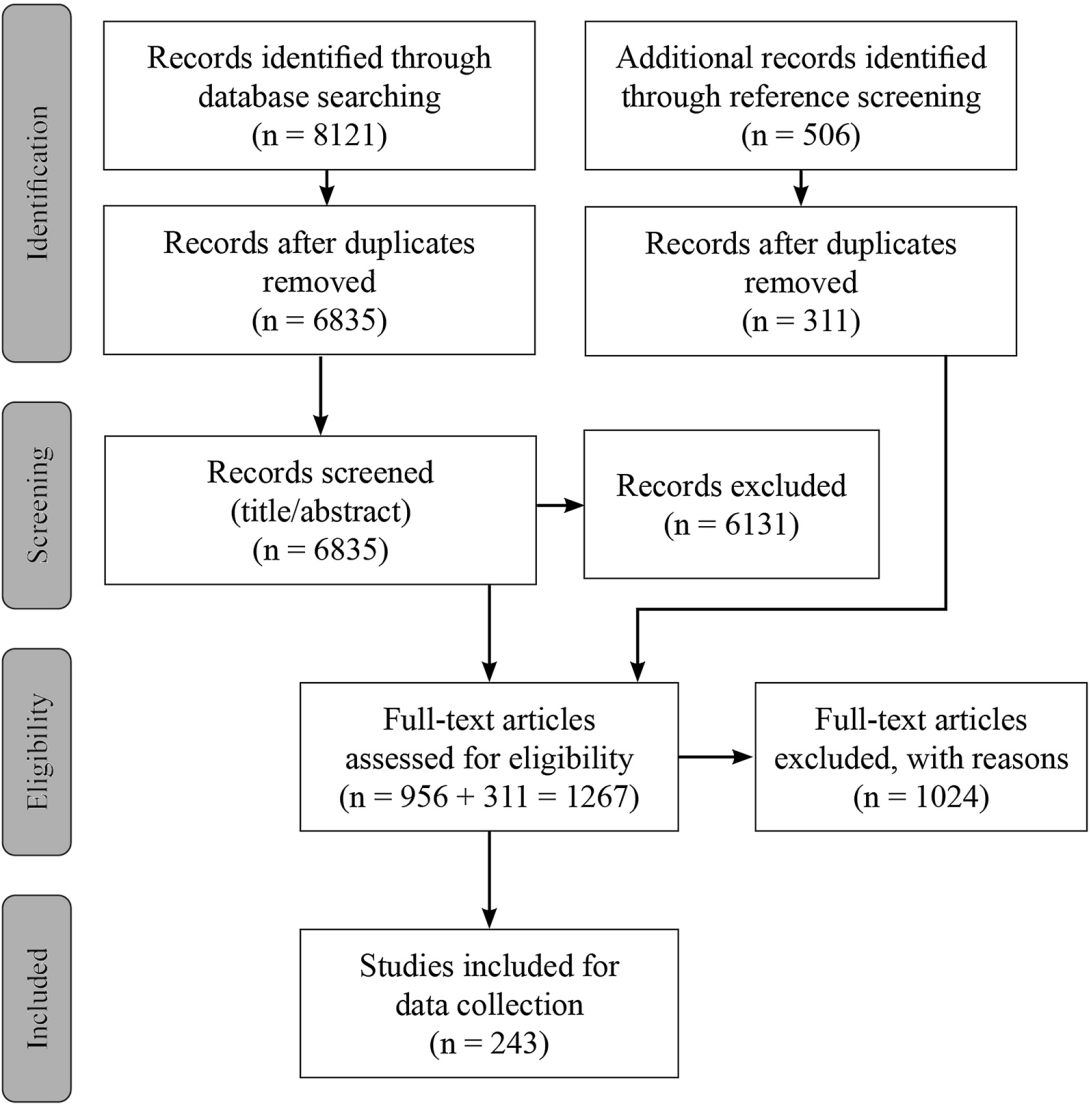
Flow chart illustrating the systematic article selection process

We identified 243 articles that met the inclusion criteria and contained at least one moral reason for or against special status for the reimbursement of OMPs. However, in 72 of all included articles (29.6%), moral reasons were merely mentioned in one paragraph or less. Figure 1 indicates the detailed results of the systematic article selection process. Reasons for exclusion on the full-text level are reported in Additional file 2. The raw data of the variables collected are available in Additional file 4.

### Distribution over time

**Fig. 2:**
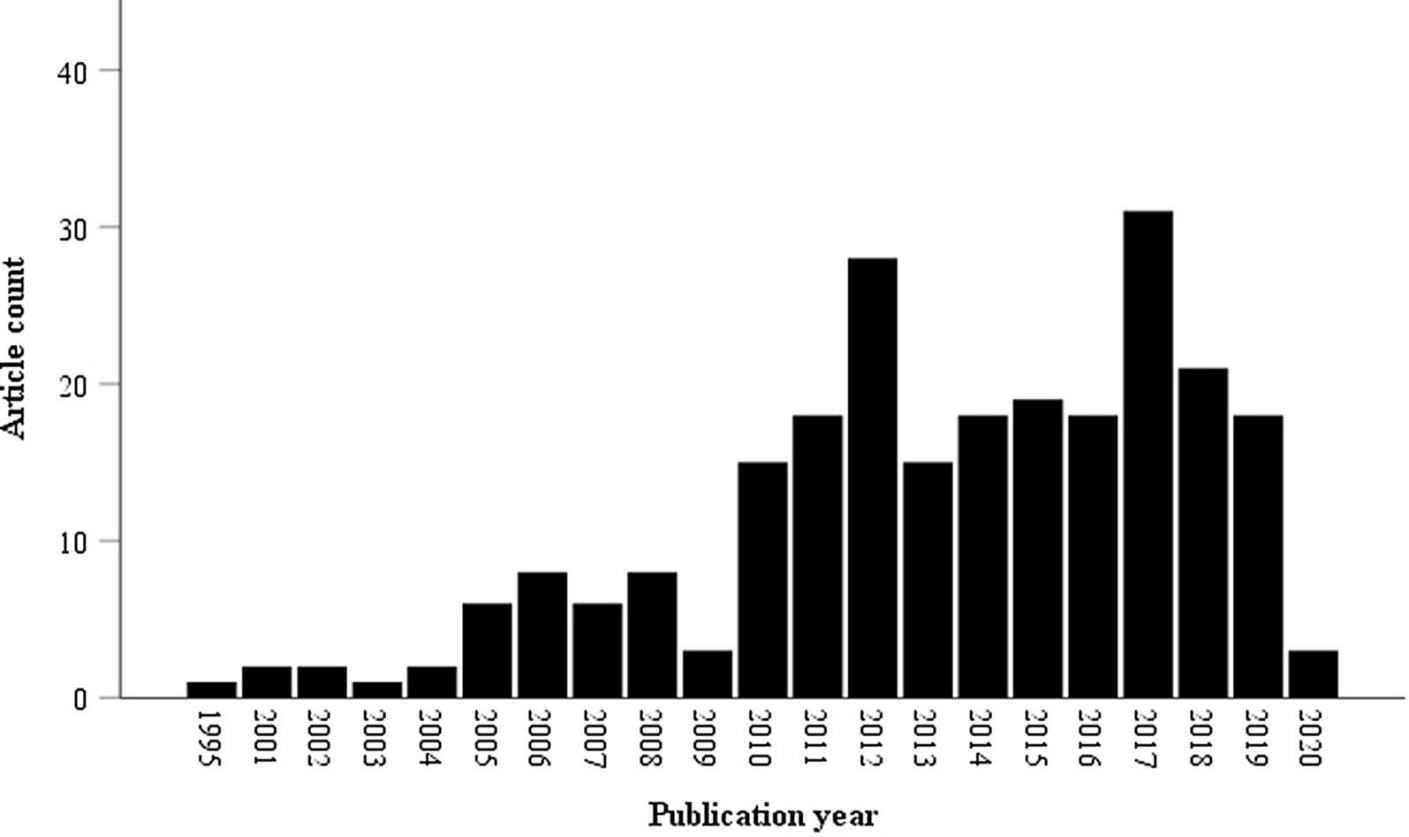
No. of included articles per year

The earliest article included was published in 1995, but all other articles were released in the years 2001 or later. Figure 2 indicates that the number of articles addressing moral reasons regarding special status for the reimbursement of OMPs increased steadily over the years, reaching a maximum of 31 articles published per year in 2017.

### Scientific disciplines

We identified a total of six scientific disciplines and added stakeholder statements from grey literature that were not assignable to any scientific discipline. Health policy was the most common discipline with a total of 103 articles (42.4%), followed by health economics (n = 49, 20.2%), philosophy and bioethics (n = 29, 11.9%), clinical medicine (n = 21, 8.6%), social sciences (n = 20, 8.2%) and law (n = 9 3.7%). Few articles included stakeholder's views, such as those from the pharmaceutical industry (n = 4, 1.6%), rare disease patients (n = 3, 1.2%), and others (n = 5, 2.0%). As Table 1 illustrates, the majority of articles from the fields of philosophy/bioethics, clinical medicine, and health economics were theoretical articles, commentaries or editorials. The health policy field applied the most variable methodology, dominated by reviews, empirical and theoretical work. In the social sciences, most articles included empirical work, especially surveys. Most stakeholder articles included policy guidelines.

**Table 1:** Frequency of represented disciplines and methodologies applied in each discipline

Discipline	Methodology													
	Theoretical/comments/editorials			Empirical		Reviews		Policy guidelines		Legal		Other		Total
Philosophy/bioethics	25 (86%)			0 (0%)		3 (10%)		1 (3%)		0 (0%)		0 (0%)		29 (100%)
Clinical medicine	12 (57%)			3 (14%)		4 (19%)		1 (5%)		1 (5%)		0 (0%)		21 (100%)
Health economics	21 (43%)			12 (24%)		16 (33%)		0 (0%)		0 (0%)		0 (0%)		49 (100%)
Health policy	31 (30%)			21 (20%)		42 (41%)		7 (7%)		1 (1%)		1 (1%)		103 (100%)
Law	2 (22%)			0 (0%)		1 (11%)		0 (0%)		6 (67%)		0 (0%)		9 (100%)
Social sciences	0 (0%)			18 (90%)		1 (5%)		0 (0%)		0 (0%)		1 (5%)		20 (100%)
Industry (stakeholder)	1 (25%)			0 (0%)		0 (0%)		3 (75%)		0 (0%)		0 (0%)		4 (100%)
Patients (stakeholder)	0 (0%)			0 (0%)		0 (0%)		3 (100%)		0 (0%)		0 (0%)		3 (100%)
Other	0 (0%)			0 (0%)		0 (0%)		3 (60%)		0 (0%)		2 (40%)		5 (100%)
Total	92 (38%)			54 (22%)		67 (28%)		18 (7%)		8 (3%)		4 (2%)		243 (100%)

### Geographical regions

Most articles focused on European countries (n = 96, 39.5%), 20 on Canada (8.2%), 19 on the United States (7.8%), 10 on Asian countries (4.1%), 8 on Australia or New Zealand (3.3%), and 3 on South American countries (1.2%). Some 41 articles (16.9%) covered more than one geographical region, while 46 articles (18.9%) specified no geographical region.

#### Overall assessment regarding special status for the reimbursement of OMPs

As indicated in Table 2, more articles drew overall conclusions in favour of the special status for OMPs than ones that drew against it. The discipline we referred to as social sciences, mainly including empirical survey studies with parts of the general population or stakeholder groups, held the highest relative number of articles arguing against special status. By contrast, in the fields of clinical medicine and law, the majority of articles argued for special status.

**Table 2:** Percentage of overall conclusion, presented separately for each discipline

Discipline	Overall assessment regarding special status				
	For special status	Against special status	Conditionally in favour	Unclear	Total
Philosophy/bioethics	13 (45%)	4 (14%)	8 (28%)	4 (14%)	29 (100%)
Clinical medicine	12 (57%)	2 (10%)	6 (29%)	1 (5%)	21 (100%)
Health economics	17 (35%)	6 (12%)	21 (43%)	5 (10%)	49 (100%)
Health policy	40 (39%)	11 (11%)	32 (31%)	20 (19%)	103 (100%)
Law	6 (67%)	0 (0%)	1 (11%)	2 (22%)	9 (100%)
Social sciences	2 (10%)	7 (35%)	10 (50%)	1 (5%)	20 (100%)
Industry (stakeholder)	2 (50%)	1 (25%)	1 (25%)	0 (0%)	4 (100%)
Patients (stakeholder)	3 (100%)	0 (0%)	0 (0%)	0 (0%)	3 (100%)
Other	2 (40%)	0 (0%)	3 (60%)	0 (0%)	5 (100%)
Total	97 (40%)	31 (13%)	82 (34%)	33 (14%)	243 (100%)

### Reasons for and against special status for the reimbursement of OMPs

**Fig. 3:**
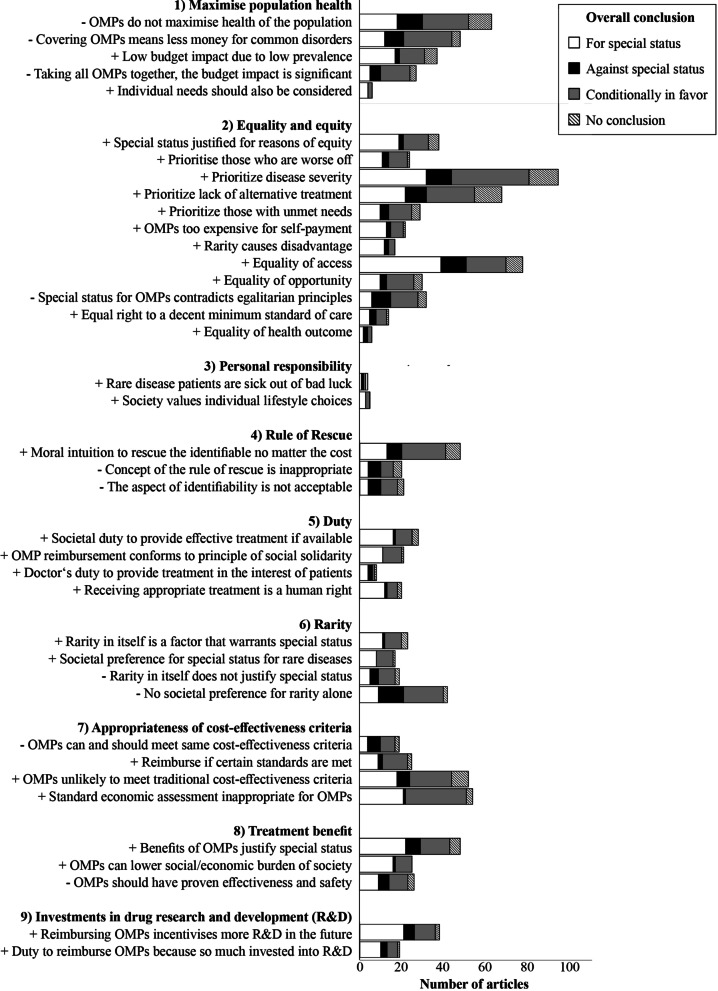
Overview of reasons for and against special status for reimbursement of OMPs. +  = reasons for special status, − = reasons against special status
  Some authors argued that reimbursing OMPs would have *positive effects on future drug development efforts* [29, 43, 52, 54, 60, 61, 75, 85, 87, 88, 96, 103, 114, 115, 118, 120, 122, 127, 128, 137, 142, 147, 169, 193, 198, 202, 205, 210, 215, 219, 220, 222, 242, 243, 248, 249, 265, 267], which would increase competition and lower prices of OMPs [60, 85, 118, 122, 147, 249, 265]. Consequently, the OMP drug development would have a positive effect for other diseases [43, 52, 54, 60, 61, 88, 115, 169, 202, 210, 242, 243], letting future generations profit from today's investments [198]. Therefore, according to some authors, the innovativeness of OMPs should be valued when considering their costs [54, 85, 114, 120, 122, 128, 141, 198, 202, 219, 220, 222].
  Furthermore, some reasoned that OMPs should be reimbursed despite their high prices because *taxpayers had already invested in their development* [45, 46, 63, 88, 112, 114, 115, 127, 128, 142, 155, 161, 231, 239, 248, 249, 259, 268, 269] through the existing incentives. Consequently, not reimbursing them would be a waste of public resources [127, 248]. Moreover, it would be wrong if patients who had contributed to successful trials could not benefit further from treatments due to costs [239, 259]. Counter-arguments to this point included that these incentives were developed for the research and development of OMPs, not for their reimbursement [112] and that the lack of cost-effectiveness would question the existing incentives rather than obligating them for reimbursement [161].

We identified 29 reasons for, and 10 reasons against special status for the reimbursement of OMPs in the scientific and grey literature and categorized them into nine categories (Fig. 3 and Additional file 5). The following section presents the content of each category with various moral reasons in detail.

1. *Maximize population health*
  The utilitarian principle to maximize overall population health is the underlying moral standard for traditional cost-effectiveness measures. Relevant to this category, we identified three broad reasons against special status for reimbursement of OMPs. First, the most commonly mentioned reason against special status for the reimbursement of OMPs (n = 63, see also Additional file 5) was that it *contradicted the principle to maximize population health* [25–27, 35–94], leading to ineffective use of resources [92] and thereby emphasizing policy makers’ obligation to use resources effectively [91]. However, 19 of the articles that mentioned this reason were generally in favour of a special status [25, 41, 46, 48, 49, 56, 57, 60, 67, 70, 72–74, 85, 87, 89, 90, 93, 94] and either refuted this reason or judged other reasons as being more important. For instance, some authors argued that *individual needs should also be considered*, not only population needs [41, 72, 91, 95, 96].
  Second, a related argument focused on the opportunity costs when reimbursing expensive OMPs, particularly that *less money would be available to treat common disorders* [35, 38, 39, 41, 45, 47, 48, 53, 62, 63, 66, 69, 73, 79, 81, 85, 88, 91–93, 97–124]. Some authors perceived this as problematic since few expensive OMP treatments would take away resources for cost-effective treatments for more patients. While some authors used this reason as an argument against special status, others more mildly called for more consideration during decision-making to weigh the benefits of OMPs against their opportunity costs. This second reason is also based on the principle of maximising population health gain but emphasizes the difference between rare and common disorders more explicitly than the first reason presented.
  Finally, differing discussions were made concerning the budget impact of OMPs. While in some articles (n = 37), authors claimed that *OMPs had a low budget impact* and could therefore be reimbursed [29, 36, 42, 44, 46, 48, 49, 52–54, 66, 74, 77, 85, 88, 103, 104, 114, 115, 118–120, 123, 125–138], others (n = 27) rejected this reason based on the claim that *all OMPs together had a significant budget impact* that would increase further in the future, since more OMPs were expected to come onto the market [35, 47, 66, 69, 73, 78, 80, 92, 100, 104, 107, 108, 110, 112, 114, 118, 122, 139–148].
2. *Equality and equity*
  Reasons related to equality and equity were discussed in many articles (n = 194, 79.8%). It became evident that the expressions of equity and equality were confounded and sometimes used interchangeably in our data. Here, we use the term “equality” in an egalitarian tradition to treat every person equally. The term “equity” is related to the idea that some compensation is allowed for ethically relevant aspects that makes people unequal, thereby allowing for prioritizing those with special needs. In some articles, authors referred to horizontal and vertical equity to make this distinction [38, 45, 74, 81, 87, 121]. Opposing views in this category concluded, for instance, that special status for *reimbursement of OMPs was incompatible with the egalitarian principle to treat each individual equally* [35, 36, 39, 42, 47, 52, 62, 70, 75, 78, 81, 85–88, 98, 100, 107, 110, 114, 132, 137, 142, 145, 149–156].
  On the other hand, an extensive body of literature argued that *a special status was justified for reasons of equity* [8, 35, 39, 46, 54, 66, 68, 73, 81, 85, 94, 107, 116, 117, 121, 122, 128, 136, 137, 152, 153, 157–164]. Reasons related to this equity principle included: First, that rare disease patients should have the *same access to treatments as all other patients should* and that this was only possible by providing OMPs even if they were expensive [5, 25, 37, 38, 40, 42–44, 51, 57, 60, 61, 63, 67, 70–72, 78–81, 87–89, 93, 95, 96, 100, 101, 103, 105, 106, 112, 115, 118, 126–129, 139, 140, 142, 151, 153, 154, 156, 157, 161, 165–194]. Any barriers to access should be “morally justifiable” [139, 183]. It was also stated that there was a general societal preference for equal access and opportunity [40, 61, 63, 79, 80, 106, 142, 151, 161, 175, 176, 182].
  Second, some authors stated that all people should have *equal opportunities for good health* [27, 35, 47, 52, 56, 66, 68, 69, 73, 81, 88, 90, 92, 93, 95, 104, 105, 109, 124, 128, 137, 142, 146, 152, 156, 173, 175, 190, 195, 196], including a “fair chance to live an autonomous and fulfilling life” [124], which would particularly prioritize young patients, as often this is the case in patients with rare diseases [90, 124, 195, 196]. Counter-arguments included that it was not feasible to reach full equality of opportunity [69], that the focus on a fulfilling life would be unfair towards the elderly or severely disabled [47], and that this proposal would be best solved in a health care lottery, which was unacceptable for Juth [105] but proposed by others as being part of a potential solution [68].
  A third and related reason for prioritizing OMPs for reasons of equity included that we should aim for *equality in health outcome*, instead of equality in access to care, which would prioritize those in worse health states, aiming at equal health and life-expectancy for everyone [35, 47, 70, 105, 173, 184]. Again, the main counter-argument concluded that this point was not specific to rare diseases and would therefore not justify special status for reimbursement of OMPs [105].
  A fourth justification for equity included the reason that everyone had the *same right to a decent minimum standard of care* [47, 49, 52, 53, 56, 63, 73, 80, 81, 90, 105, 114, 115, 196]. This line of argument, also called sufficientariarism in the literature [35, 47, 105], was often referred to as a form of prioritarianism where those that are prioritized have more difficulties reaching this decent minimum standard of care. However, Gross [196] argued that first, access to primary health care for all should be ensured before considering reimbursement of OMPs. Others stated that sufficientariarism was inappropriate to judge for special status in OMPs because it was unclear what the “decent minimum” encompassed [90] and the concept would not necessarily prioritize OMPs [105].
  Finally, prioritizing OMPs for reasons of equity was justified by the reason that rare disease patients were *worse off compared to others* [27, 28, 35, 38, 41, 46, 47, 69–71, 73, 74, 79, 87, 101, 105, 108, 109, 122, 152, 161, 196–198], which was also framed as a societal preference [27, 46, 73, 74, 79, 122, 161, 198]. The issue of *how* rare disease patients were worse off than others was discussed extensively, including (1) disease severity; (2) unmet needs; (3) lack of alternative treatments; (4) the high-cost treatments not allowing for self-payment; and (5) the general disadvantages of rare disease patients. In the following paragraphs, these five aspects will be reviewed in greater detail.
  (1) Many authors called for granting special status as OMPs treated *severe conditions* [24–27, 35, 37, 38, 40, 42–46, 49, 51–57, 59, 63, 69, 70, 72–75, 78–80, 83, 86–90, 92, 93, 95, 102, 104, 106, 107, 109, 112, 114, 115, 117–119, 122–124, 129, 130, 132, 135, 139, 142, 144, 150, 156, 157, 161, 167, 176–178, 184, 192, 193, 199–219], where this disease severity was a factor of societal preference. Some authors generally referred to disease severity as an important criterion for reimbursement decisions, irrespective of whether a disease was rare or common [27, 46, 49, 57, 70, 83, 87, 89, 102, 117, 120, 139, 141, 202, 208, 218, 219]. As stated by Medic et al. [87], the extent that rarity contributes to perceived severity in decision-makers’ eyes is unclear. Consequently, several authors specified that disease severity was not an argument specific to OMPs [44, 75, 86, 88, 104, 112, 117, 142, 150, 205] and not all rare diseases were severe or life-threatening [52, 55, 63, 130], thereby concluding that disease severity was not a good criterion to justify special status. Moreover, disease severity would need a unified method of measurement to serve as an objective reimbursement criterion [208]. In the considered body of literature, diseases were considered severe if, for instance, they were “chronic, deliberating and associated with reduced life expectancy” [53] or were associated with severe pain [212]. (2) Another feature justifying special status for the reimbursement of OMPs were *unmet needs*. Authors argued that in the case of unmet needs, it was unfair not to cover those treatments [24, 36, 57, 69, 75, 77, 79, 87, 89, 99, 102, 114, 116, 122, 129, 130, 136, 178, 184, 189, 202, 203, 205, 208, 220–224]. However, Sandman and Hofmann [184] found the concept of unmet needs unsustainable in this context and recommended instead considering disease severity. (3) One extensively discussed reason (n = 68) related to unmet need was the *lack of alternative treatment* [8, 24, 37, 44–46, 51, 54, 56, 60, 62, 64, 70, 75, 77–79, 83, 84, 87, 96, 111, 112, 114–116, 122, 123, 128–130, 135, 139, 141, 142, 147, 150–152, 161–163, 167, 174, 177, 178, 184, 193, 199–203, 206, 208, 210–215, 217, 219, 223, 225–228]: Since OMPs often represented the only hope for patients suffering from rare diseases, which justified their special status [70]. Relatedly, some studies also found a societal preference for covering drugs for diseases without treatment alternatives [45, 51, 79, 115, 213, 217]. However, some other authors countered that supportive or palliative care was always an available alternative [75, 83, 111, 112, 142, 184]. These reasons of disease severity, unmet needs or the lack of alternative treatments were often mentioned in the same paragraph (n = 36), and were usually presented as features that needed to be fulfilled to justify special status [24, 37, 44–46, 51, 54, 56, 75, 78, 79, 83, 87, 112, 122, 123, 129, 135, 139, 142, 150, 161, 163, 177, 178, 193, 199–201, 206, 210–213, 217, 219]. A common counter-argument for disease severity, unmet needs and the lack of alternative treatment was that they were not specific to rare diseases and could also be a feature of certain common diseases [35, 36, 44, 75, 86, 88, 104, 105, 111, 112, 117, 142, 150, 205, 224, 225]. (4) An argument that specifically draws on the feature of rarity was that *rare disease patients were historically disadvantaged due to the rarity of their diseases* and therefore generally worse off [35, 43, 73, 88, 94, 97, 108, 117, 152, 165, 173, 175, 179, 210, 229–231]. These disadvantages include: the lack of drug development efforts before incentives were installed [97, 117, 173, 175, 229]; disadvantages in traditional cost-effectiveness procedures [43], as Quality-Adjusted Life Years (QALY) measures would disadvantage people with chronic disabilities [73, 210]; the lack of public awareness; and difficulties in collecting effectiveness evidence for OMPs [165]. (5) A final justification for why rare disease patients were worse off is that *OMPs were too expensive to be paid by patients themselves* and therefore should be reimbursed [46, 51, 61, 116, 118, 123, 133, 138, 147, 161, 163, 183, 199–201, 204, 208–210, 232–234]. It was also argued that rare disease patients already carried a substantial financial burden as compared to the general population [209, 210, 233], including indirect costs for transportation to specialized facilities or the inability of family caregivers to be in full-time employment [123].
3. *Personal responsibility*
  Several authors mentioned that *society values the role of individual lifestyle choices*, such as smoking, diet or physical activity [73, 74, 88, 124, 208]. In some societies, the willingness to cover expensive treatments for diseases that are negatively influenced by individual lifestyle choices seems to be lower [73, 74, 124, 217]. For example, this was noted by the National Institute for Clinical Excellence (NICE) Citizens Council report [88]. In line with this argument, the reason given for special status of the reimbursement of OMPs was that rare disease patients were *sick out of bad luck* and could not be held responsible for their situation [47, 137, 177, 231].
4. *Rule of rescue*
  A considerable body of literature (n = 48) discussed the controversial Rule of Rescue, which justifies the *rescue of endangered lives of identifiable patients by moral intuition*, no matter the cost [24–26, 29, 35, 38, 41, 42, 47–49, 52, 53, 58, 60, 63, 64, 67, 70, 71, 73, 74, 77, 77, 81–83, 91, 104, 108, 109, 114, 116, 123, 125, 139, 151, 153, 175, 181, 187, 196, 217, 230, 235–238]. Mass media coverage [151, 236–238], the moral instincts of physicians [236] as well as society in general [175, 217, 237] were said to support the Rule of Rescue. The benefit of reimbursement was argued to be particularly high because, to be covered under the Rule of Rescue, OMPs must be life-saving or at least be able to significantly improve the quality of life [108]. Authors reasoned that rare disease patients were easily identifiable due to the rarity of their conditions, which would make it morally difficult to deny available treatment, even if it was costly [70, 91, 104, 108, 139, 181], thereby deriving from this feature of identifiability a moral obligation to rescue for reasons of social relatedness [26, 91]. It was also argued that the rarity of the conditions would limit the budget impact when applying the Rule of Rescue [24, 83, 108, 123, 153].
  Counter-arguments to using the Rule of Rescue for resource allocation decisions in the context of OMPs included that the concept was *inappropriate* as its consistent application on a population level was not possible [46, 61, 75, 83, 109, 111, 123, 143, 151, 231] and would even be discriminatory against those not in an immediate life-threatening situation [107]. The Rule of Rescue, some argued, was therefore unsustainable as a policy guiding principle [58, 82, 83, 108, 109]. Moreover, many aspects of the Rule of Rescue were said not to be specific to rare diseases [62, 66, 75, 83] and not all OMPs would meet the criteria of saving lives from immediate life-threatening illnesses [92, 196]. It was also criticized that the Rule of Rescue was based on emotion thus was incompatible with rational decision-making [40, 83, 111], failed to maximise population health [41, 109], and that its aim of saving lives was inappropriate since death was unavoidable for human beings [26, 83]. Finally, the aspect of *identifiability* was subject to broad criticism, as this reliance on identifiability was argued to be inequitable and unfair [40, 41, 66, 72, 81, 91, 92, 108, 109, 111, 114, 196, 230], morally irrelevant [91, 105] and “merely a matter of time and perspective” [111]. Finally, it was feared that relying on identifiability would be a bottomless pit concerning budget impact [105, 109].
5. *Duty*
  Several reasons connected to a sense of duty to serve those affected by rare diseases. First, it was argued that *society had a duty to not abandon rare disease patients* and to provide effective treatment if available [25, 56, 68, 69, 72, 79, 81, 88, 90, 107, 115, 122, 124, 127, 136, 137, 157, 168, 173, 188, 189, 196, 218, 239–243]. The principle of social justice would demand that everyone was treated with dignity and respect [56, 81, 127, 168, 189]. This would conform to the *principle of social solidarity*, which is the moral basis of publicly funded health care systems that reflect societal compassion to those affected by rare diseases [27, 48, 73, 88, 109, 122, 124, 126, 136, 208, 215, 229, 244]. It was also argued that *receiving appropriate treatment was a human right*, that included the right to healthcare [25, 44, 46, 66, 70, 73, 76, 93, 101, 114, 125, 128, 143, 147, 159, 178, 244–247], even if OMPs were expensive [66, 70, 93, 159, 244, 245].
  A final duty-based reason was that it was every *doctor's duty to provide treatment in the interest of patients*, according to the Principle of Beneficence [25, 26, 43, 60, 93, 107, 137, 236], which was then countered by the argument that it was also a doctor's duty to avoid overly expensive treatments [43].
6. *Rarity*
  The influence of rarity as a property that directly justifies special status for the reimbursement of OMPs was debated controversially. Those in favour stated that *rarity in itself was a factor that warranted special status* [35, 45, 73, 74, 87, 92, 103, 109, 118, 122, 127, 135, 155, 157, 161, 206, 208, 214, 221, 245, 246, 248, 249] because rarity made evidence collection more challenging [73] and necessarily led to high prices [35, 74, 87, 103, 109, 122, 127, 135, 155, 208, 214, 248, 249]. By contrast, others argued that disease prevalence was a morally arbitrary distinction for reimbursement decision-making [35, 43, 62, 100, 105, 114, 117, 120, 130, 148, 152, 156, 182, 208, 219, 224, 246, 250].
  A *societal preference for prioritizing rarity* was substantiated by notions that payers already made exceptions when reimbursing OMPs [35, 46, 87, 202, 215] and that laws incentivising OMP development demonstrated a societal willingness to pay [152, 173]. Part of the NICE Citizens' Council [88], as well as a European population survey on rare diseases [251], reported in favour of prioritizing rare disease patients in resource allocation.
  Since 2010, several country-specific population surveys assessing the societal preference for rarity were published. In contrast to the previously mentioned reports, they mostly concluded that there was *no societal preference for rarity alone* [24, 26, 27, 35, 38, 39, 42–46, 63, 73, 75, 78–80, 87, 92, 93, 99, 111, 112, 114, 116, 132, 151, 157, 161, 167, 173, 182, 198, 205, 208, 212, 213, 217, 252–254], but did find general preferences for considering equity in healthcare resource allocation, including preferential reimbursement for severe diseases without available alternatives [24, 44, 63, 80, 116, 167, 182, 205, 212, 213, 254]. Similarly, it was shown that healthcare professionals were not generally prioritizing rarity [43, 182]. Nevertheless, population surveys face important methodological challenges, including framing effects, unstable societal preferences stemming from low public engagement with the issue, and the unwillingness of study participants to adhere to the frame that finite resource must be redistributed between rare and common diseases [99].
7. *Appropriateness of cost-effectiveness criteria*
  Some authors argued against special status, stating that *OMPs could and should meet the same cost-effectiveness criteria as any other drug* [38, 45, 53, 62, 73, 78, 82, 85, 90, 104, 105, 111, 114, 127, 134, 135, 142, 150, 194]. However, other authors mentioned that *OMPs were unlikely to meet traditional cost-effectiveness criteria* [24, 35, 36, 38, 41, 43, 45, 54, 57, 59, 62, 64, 65, 68, 72, 73, 75, 87, 89, 109, 116, 118, 120, 122, 128, 140, 143, 144, 151–153, 155–158, 162, 178, 196, 199–201, 206, 208, 220, 221, 224, 244, 252, 255–258], which would leave many rare disease patients without any treatments. Moreover, the *standard cost-effectiveness analysis was argued to be inappropriate* or at least not optimal for assessing the value of OMPs since it did not take equity or other societal values into account [27, 38, 54, 72, 75, 132, 152, 178, 192], thereby disrespecting societal preferences [41, 59, 72, 101, 135, 144, 145, 147, 208, 259–263] and the idea of a solidaristic health care system [145, 264]. Decision-makers should also take into account the difficulties in obtaining the necessary evidence [24, 36, 62, 64, 72, 122, 178, 208, 220, 221, 257]. In addition, it was argued that cost-effectiveness exceptions were also made in other situations, such as in rescuing mountaineers or transplant operations [46, 173].
  In the middle ground between the arguments calling for the same cost-effectiveness criteria and those calling for reimbursing OMPs at any cost, several authors argued that OMPs, too, should *meet certain cost-effectiveness standards*, but that these should adapt to the particular situations of rare diseases and OMPs [47, 54, 72, 82, 87, 88, 92, 95, 98, 104, 109, 114, 120, 128, 129, 136, 141, 150, 151, 174, 202, 208, 217, 224, 244]. For instance, it was suggested to use additional criteria for assessing the reimbursement of OMPs [29, 45, 114], such as the budget impact of a drug [54, 87, 114, 120, 120, 141, 150, 174, 200, 202, 208, 224, 244].
8. *Treatment benefit*
  Another line of argument was that a special status was justified if *OMPs provided a high benefit* [24, 26, 43–47, 63, 72, 78, 79, 87, 88, 93, 104, 107, 109, 112, 114–116, 120, 125, 127–132, 136, 152, 153, 161, 167, 178, 184, 195, 202, 203, 208, 212, 214–216, 249, 260, 265, 266]. The nature of this benefit, however, was defined differently [208]: some authors referred to characteristics such as life-saving [47, 93, 109, 115, 127, 128, 132, 136, 152, 153] or curative treatments [78, 79, 104, 125, 129, 214, 215, 266]; others mentioned the ability to restore societal functioning [79, 109, 130, 195, 249, 265]. For some, an improved quality of life [26, 79, 88, 107, 120, 132, 136, 152, 161, 202, 266] or even just a stabilization [88, 125, 152, 212, 249] was enough of a benefit for OMPs to be covered. It was also argued that *OMPs contributed to lowering the social and economic burdens* that rare diseases imposed on society [54, 59, 79, 93, 94, 96, 101, 114, 115, 118, 122, 123, 131, 140, 168, 174, 195, 210, 211, 215, 216, 249, 261, 265, 266] and that these costs should be taken into account when assessing the effectiveness of OMPs. Some authors stated that, despite the difficulties in obtaining evidence combined with the additional development costs, it was crucial that these reimbursed OMPs had *proven effectiveness and safety* [36, 41, 49, 62, 68, 72, 78, 79, 88, 114, 116, 128, 129, 136, 141, 142, 174, 191, 193, 194, 202, 207, 208, 229, 241, 257], thereby calling for alternative ways of evidence creation (e.g. real-world evidence, dose–response studies, and expert opinions) [68, 79, 141, 193, 194, 229] or defining it under the manufacturer's obligation to deliver as much evidence as possible [36, 72].
9. *Investments in OMP research and development*

## Discussion

In this study, we reviewed moral reasons for and against special status for the reimbursement of OMPs from a multidisciplinary perspective. As indicated, the problem is manifold and not only discussed among ethicist, but also considered extensively in the fields of health policy and health economics. On the normative-theoretical level, there is no clear-cut solution to whether or not OMPs should receive special status for their reimbursement, as different moral theories come to different, sometimes opposite conclusions. This underlines the importance of analysing the problem from a broader, multidisciplinary perspective.

Currently, OMP reimbursement is often based on rules of exception in publicly-funded healthcare systems. This is problematic from a clinical perspective because it leaves the responsibility of whether and when to reimburse OMPs on clinicians and healthcare insurances raising problems related to equity and fairness. The issue is further complicated for clinicians by differing perspectives when considering the well-being of patients. From a public health perspective, it may be necessary to impose cost constraints on individual patients to not abandon others; from the perspective of an individual patient affected by a rare disease, however, it may be ethically challenging to deny an available treatment merely due to cost constraints. These two perspectives are also represented in the various reasons identified: while reasons that focused on the individual patient, such as the duty to care or the rule of rescue, all argue in favour of special status, several reasons that focused on the societal level argue against special status (for example those whose reasons were relevant to maximising population health). This demonstrates the dilemma clinicians and health insurances are facing when having to prescribe OMPs based on exceptions. It is, therefore, crucial to identify fair, consistent and equitable rules for when to prescribe OMPs. In publicly funded health care systems, this lies in the responsibility of HTA agencies. In the following, we will discuss the connection between economic and moral considerations as well as the heterogeneous characteristics of rare diseases and OMPs in light of their implications for HTA agencies and other bodies responsible for defining reimbursement rules.

Because traditional standards for reimbursement in publicly funded health care systems are based on cost-effectiveness evaluations, giving OMPs a special status in this process requires additional reasoning. This is reflected in the literature, as the review identified more articles and a higher number of reasons in favour of special status. Cost-effectiveness has a strong economic component. However, as utilitarianism demonstrates, optimizing the allocation of health care costs also has a moral component. Indeed, the relevance of economic criteria in health care resource allocation roots in utilitarianism. Most of the reasons detected against special status are based on or connected to utilitarianism, whereas many reasons in favour of special status aim to overcome economic considerations due to alternative moral concepts and theories. Consequently, health economics plays a crucial role even in the moral assessment of HTA decision-making and economic considerations should neither be considered the gold standard nor be neglected in HTA decision-making in the context of OMPs.

Many reasons based their arguments for or against special status on disease prevalence. The question of whether rarity justifies special status demonstrates a binary understanding of rarity: a disease is either rare or common. This is problematic for two reasons. First, it oversimplifies the issue at stake since disease prevalence is not the only factor considered in HTA assessments. Second, the focus on disease prevalence also reverts to the question of how to define rare diseases. They are highly heterogeneous by being, for instance, of differing severity, having a varying prevalence in different regions of the world, and composing varying budget implications for treatment options. This makes it difficult, if not impossible, to find a one-fits-all solution for OMP reimbursement. Rather, ways to address this heterogeneity rationally should be identified with all the stakeholders involved. Factors representing this heterogeneity from a moral perspective include, for instance, disease severity, availability of alternative treatment options, innovativeness of OMPs, or the budget impact. Moreover, considering disease prevalence as a continuous rather than a binary variable and using alternative evidence creation, such as real-world evidence or n-of-1 trials [270], might support efforts for a more fine-grained and heterogeneous, yet fair and consistent examination of OMP HTAs. Decision frameworks using more flexible and variable approaches, such as the EVIDEM framework [79, 164] or the Multi-Criteria Decision Analyses (MCDA) [50, 154, 256], represent promising alternatives implementing these factors but might need further refinement to optimize all factors included. The advantages of these frameworks include that they can be applied to all drugs, which avoids the issue of the special status of OMP reimbursement at least from a procedural perspective while allowing for a more fine-grained distinction between the two groups “OMPs” versus “non-orphan drugs”. Due to the increasing decision-making complexity, computer-assisted HTA could support parts of the decision-making process and should be evaluated in the future [271].

Future research on the reimbursement of OMPs should also focus on crowdfunding as alternative funding schemes from both empirical and normative perspectives, as the rationale of crowdfunding is closely linked to the controversially debated Rule of Rescue. Moreover, existing studies investigating the views of the general population on OMP reimbursement come with methodological shortcuts, as it is difficult to assess the preferences in healthcare resource allocation amid such a complex ethical challenge. Survey studies, for instance, might be subject to biases in the framing of questions or the choice of scenarios [272]. As the opinion of the majority of tax payers is commonly used to reason for or against special status of OMPs, this warrants further effort in identifying additional, reliable ways to empirically assess public preferences concerning OMP reimbursement.

### Limitations

For this review, we followed the established methodology of systematic reviews of reasons. Yet, the study still faces several limitations. First, parts of the findings, including the reasons for or against special status for the reimbursement of OMPs, were assessed in an inductive, qualitative manner. However, a reliability test that is usually performed for quantitative content analyses to ensure consistency and transparency in data collection, was not feasible due to the variety of different reasons and the complexity of the analysis. This has already been acknowledged in studies applying a similar methodology, and we attempted to overcome this issue by working and discussing the collection of data in a research team, but it should be noted that any presented frequencies should be viewed descriptively and in relative importance to each other. It was also for this reason that we refrained from any statistical analysis. A second limitation is that, due to the complexity of the topic, it was challenging to identify all of the relevant articles from all relevant disciplines. We purposefully used a broad search term in a variety of databases and additionally screened the references of the included articles, but it might still be possible that relevant articles are missing. Third, the systematic screening of grey literature was challenging because grey literature databases did not retrieve any results, thus many grey literature reports were identified through individual online searches or reference screening. Moreover, the strong focus on OMP reimbursement might be the reason for the predominance of articles within the European context and relatively low representation of the United States. While the analysis was not limited to the European context, this analysis targets the focus on reimbursement, which is a typical feature of publicly funded health care systems. This might narrow down the reasons identified, and might be the reasons behind, for instance, the underrepresentation of libertarian arguments. Moreover, we did not assess the adequacy and quality of reasons but rather condensed them as they were found in the literature. We also did not collect data on how reasons within one article were interconnected and we counted reasons that were cited from other authors as distinct reason mentions. However, by portraying the reasons in context to the overall conclusion of articles, we still provide some context within which the reasons were mentioned.

## Conclusion

On the normative-theoretical level, there is no clear-cut solution to whether or not OMPs should receive special status for their reimbursement. However, for reasons of fairness, it is crucial to identify consistent and equitable rules for when to prescribe OMPs. The heterogeneity of characteristics of both rare diseases and OMPs makes it difficult, if not impossible, to find a one-fits-all solution for OMP reimbursement. Therefore, the binary perspective of whether or not OMPs should be granted special status seems to oversimplify the issue. Yet, moral reasonings mostly focus on such a binary perspective. The authors suggest that it might help address the issue of OMP reimbursement if the scientific debate focused more on how the important variabilities of different OMPs concerning target population, cost-effectiveness, level of evidence, or mechanism of action could be meaningfully addressed and implemented in HTAs. We further suggest that computer-assisted decision aids might be helpful supporters to rationalize and simplify the work of HTA agencies. However, those rely on robust empirical evidence, and this review revealed a number of under-researched areas that need to be addressed in the future, including the views of patients and caregivers, robust empirical assessment of population surveys, or the normative and empirical assessment of crowdfunding OMPs.

## Supplementary Information

**Additional file 1**. **Database search strategy**. Including database-specific search algorithms and search details.

**Additional file 2**. **Article selection: excluded articles**. Specifies how many articles were excluded on the full text screening stage for what reasons.

**Additional file 3**. **Codebook**. Includes variables and categories used for systematic data extraction.

**Additional file 4**. **Raw data**. Includes all data extracted from included articles.

**Additional file 5**. **Specification of articles and moral reasons**. Includes a table as a supplementary to Figure 3, specifies which articles contain what moral reasons.

## Data Availability

The datasets supporting the conclusions of this article are included within the article and its additional files.

## Abbreviations

HTA: Health Technology Assessment
MCDA: Multi-criteria decision analysis
OMP: Orphan medicinal product
RQ: Research question
