# Supplementary material for: A systematic review of moral reasons on orphan drug reimbursement
Source: Orphanet J Rare Dis. 2021 Jun 30;16:292. doi: 10.1186/s13023-021-01925-y (PMC8247078; doi:10.1186/s13023-021-01925-y)
Supplement: Supplementary file 1 — Additional file 1. Database search strategy. Including database-specific search algorithms and search details. [file 13023_2021_1925_MOESM1_ESM.pdf]

Additional file 1

Database search strategy

EMBASE (via Elsevier)

15 April 2020, 4078 hits

('rare disease'/exp OR 'rare disease':ti,ab OR 'rare diseases':ti,ab OR 'orphan disease'/exp OR 'orphan disease':ti,ab OR 'orphan diseases':ti,ab OR 'orphan drug'/exp OR 'orphan drug':ti,ab OR 'orphan drugs':ti,ab OR 'orphan product':ti,ab OR 'orphan medicine':ti,ab OR 'orphan medicines':ti,ab OR 'orphan medicinal product':ti,ab OR 'orphan medicinal products':ti,ab OR 'orphan designation':ti,ab OR 'orphan designations':ti,ab OR 'orphan-designated':ti,ab OR 'orphan designated':ti,ab OR 'medicinal products for rare diseases':ti,ab) AND ('health care access'/exp OR 'reimbursement'/exp OR 'reimburse':ti,ab OR 'reimbursement':ti,ab OR 'refund':ti,ab OR 'refunding':ti,ab OR 'funding':ti,ab OR 'insurance coverage':ti,ab OR 'healthcare coverage':ti,ab OR 'resource allocation'/exp OR 'priority setting'/exp OR 'priority setting':ti,ab OR 'priority-setting':ti,ab OR 'health care rationing':ti,ab OR 'healthcare rationing':ti,ab OR 'health care policy'/de OR 'health economics'/exp OR 'policy making':ti,ab OR 'policy-making':ti,ab OR 'health policy':ti,ab OR 'health care policy':ti,ab OR 'healthcare policy':ti,ab OR 'health economics':ti,ab)

Note: No filter, unlimited time span

New Pubmed

15 April 2020, 899 hits

("Rare Diseases"[Mesh] OR rare disease[tiab] OR rare diseases[tiab] OR orphan disease[tiab] OR orphan diseases[tiab] OR "Orphan Drug Production"[Mesh] OR orphan drug[tiab] OR orphan drugs[tiab] OR orphan product[tiab] OR orphan medicine[tiab] OR orphan medicines[tiab] OR orphan medicinal product[tiab] OR orphan medicinal products[tiab] OR orphan designation[tiab] OR orphan designations[tiab] OR orphan-designated[tiab] OR orphan designated[tiab] OR medicinal products for rare diseases[tiab]) AND ("Reimbursement Mechanisms"[Mesh] OR "Insurance, Health, Reimbursement"[Mesh] OR reimburse[tiab] OR reimbursement[tiab] OR refund[tiab] OR refunding[tiab] OR funding[tiab] OR "Insurance Coverage"[Mesh] OR insurance coverage[tiab] OR healthcare coverage[tiab] OR "Health Services Accessibility"[Mesh] OR "Resource Allocation"[Mesh] OR Priority-setting[tiab] OR Priority setting[tiab] OR Health care rationing[tiab] OR healthcare rationing[tiab] OR "Policy Making"[Mesh] OR "Health Policy"[Mesh] OR health policy[tiab] OR health policies[tiab] OR policy making[tiab] OR policy-making[tiab] OR "Economics, Medical"[Mesh] OR health economics[tiab] OR medical economics[tiab])

Additional file 1

Note: No filter, unlimited time span

CINAHL

15 April 2020, 407 hits

((MH "Rare Diseases") OR TI "rare disease" OR AB "rare disease" OR TI "rare diseases" OR AB "rare diseases" OR TI "orphan disease" OR AB "orphan disease" OR TI "orphan diseases" OR AB "orphan diseases" OR (MH "Drugs, Orphan") OR (MH "DRUGS, INVESTIGATIONAL") TI "orphan drug" OR AB "orphan drug" OR TI "orphan drugs" OR AB "orphan drugs" OR TI "orphan product" AB "orphan product" OR TI "orphan medicine" OR AB "orphan medicine" OR TI "orphan medicines" OR AB "orphan medicines" OR TI "orphan medicinal product" OR AB "orphan medicinal product" OR TI "orphan medicinal products" OR AB "orphan medicinal products" OR TI "medicinal products for rare diseases" OR AB "medicinal products for rare diseases" OR TI "orphan designation" OR AB "orphan designation" OR TI "orphan designations" OR AB "orphan designations" OR TI "orphan designated" OR AB "orphan designated" OR TI "orphan-designated" OR AB "orphan-designated") AND ((MH "Insurance, Health, Reimbursement+") OR TI "reimburse" OR AB "reimburse" OR TI "reimbursement" OR AB "reimbursement" OR TI "refund" OR AB "refund" OR TI "refunding" OR AB "refunding" OR TI "funding" OR AB "funding" OR TI "insurance coverage" OR AB "insurance coverage" OR TI "healthcare coverage" OR AB "healthcare coverage" OR (MH "Health Services Accessibility") OR (MH "Health Resource Allocation") OR (MH "Health Resource Utilization") OR (MH "Healthcare Disparities") OR TI "Priority-setting" OR AB "priority-setting" OR TI "priority setting" OR AB "priority setting" OR TI "health care rationing" OR AB "health care rationing" OR TI "healthcare rationing" OR AB "healthcare rationing" OR (MH "Policy Making") OR (MH "Health Policy") OR TI "health policy" OR AB "health policy" OR TI "health policies" OR AB "health policies" OR TI "policy making" OR AB "policy making" OR TI "policy-making" OR AB "policy-making" OR (MH "Economic Value of Life") OR TI "health economics" OR AB "health economics" OR TI "medical economics" OR AB "medical economics")

Note: No filter, unlimited time span

Web of Science

15 April 2020, 745 Hits, Searched in "Topic" in WoS Core Collection:

("rare disease" OR "rare diseases" OR "orphan disease" OR "orphan diseases" OR "orphan drug" OR "orphan drugs" OR "orphan product" OR "orphan products" OR "orphan medicine" OR "orphan medicines" OR "orphan medicinal product" OR "orphan medicinal products" OR "medicinal products

for rare diseases" OR "orphan designation" OR "orphan designations" OR "orphan-designated" OR "orphan designated") AND (reimburse OR reimbursement OR refund OR refunding OR funding OR "insurance coverage" OR "healthcare coverage" OR "priority-setting" OR "priority setting" OR "health care rationing" OR "healthcare rationing" OR "health policy" OR "health policies" OR "policy making" OR "policy-making" OR "health economics" OR "medical economics")

Note: **Timespan:** All years. **Indexes:** SCI-EXPANDED, SSCI, A&HCI, CPCI-S, CPCI-SSH, BKCI-S, BKCI-SSH, ESCI, CCR-EXPANDED, IC. No filter.

Philosopher's Index (with full text, via EBSCOhost)

15 April 2020, 5 hits

("rare disease" OR "rare diseases" OR "orphan disease" OR "orphan diseases" OR "orphan drug" OR "orphan drugs" OR "orphan product" OR "orphan products" OR "orphan medicine" OR "orphan medicines" OR "orphan medicinal product" OR "orphan medicinal products" OR "medicinal products for rare diseases" OR "orphan designation" OR "orphan designations" OR "orphan-designated" OR "orphan designated") AND (reimburse OR reimbursement OR refund OR refunding OR funding OR "insurance coverage" OR "healthcare coverage" OR "priority-setting" OR "priority setting" OR "health care rationing" OR "healthcare rationing" OR "health policy" OR "health policies" OR "policy making" OR "policy-making" OR "health economics" OR "medical economics")

Note: No filter, unlimited time span

HeinOnline Full Text search in Law Journal Library

15 April 2020, 1754 hits

("rare disease" OR "rare diseases" OR "orphan disease" OR "orphan diseases" OR "orphan drug" OR "orphan drugs" OR "orphan product" OR "orphan products" OR "orphan medicine" OR "orphan medicines" OR "orphan medicinal product" OR "orphan medicinal products" OR "medicinal products for rare diseases" OR "orphan designation" OR "orphan designations" OR "orphan-designated" OR "orphan designated") AND (reimburse OR reimbursement OR refund OR refunding OR funding OR "insurance coverage" OR "healthcare coverage" OR "priority-setting" OR "priority setting" OR "health care rationing" OR "healthcare rationing" OR "health policy" OR "health policies" OR "policy making" OR "policy-making" OR "health economics" OR "medical economics")

Note: No filter, unlimited time span

Additional file 1

Swissbib

16 April 2020, 68 hits

("rare disease" OR "rare diseases" OR "orphan disease" OR "orphan diseases" OR "orphan drug" OR "orphan drugs" OR "orphan product" OR "orphan products" OR "orphan medicine" OR "orphan medicines" OR "orphan medicinal product" OR "orphan medicinal products" OR "medicinal products for rare diseases" OR "orphan designation" OR "orphan designations" OR "orphan-designated" OR "orphan designated") AND (reimburse OR reimbursement OR refund OR refunding OR funding OR "insurance coverage" OR "healthcare coverage" OR "priority-setting" OR "priority setting" OR "health care rationing" OR "healthcare rationing" OR "health policy" OR "health policies" OR "policy making" OR "policy-making" OR "health economics" OR "medical economics")

Note: No filter, unlimited time span

Grey literature

Opengrey (www.opengrey.eu)

16 April 2020, 4 hits

("rare disease" OR "rare diseases" OR "orphan disease" OR "orphan diseases" OR "orphan drug" OR "orphan drugs" OR "orphan product" OR "orphan products" OR "orphan medicine" OR "orphan medicines" OR "orphan medicinal product" OR "orphan medicinal products" OR "medicinal products for rare diseases" OR "orphan designation" OR "orphan designations" OR "orphan-designated" OR "orphan designated") AND (reimburse OR reimbursement OR refund OR refunding OR funding OR "insurance coverage" OR "healthcare coverage" OR "priority-setting" OR "priority setting" OR "health care rationing" OR "healthcare rationing" OR "health policy" OR "health policies" OR "policy making" OR "policy-making" OR "health economics" OR "medical economics")

Note: No filter, unlimited time span

SSOAR

16 April 2020, no results

Institutions/patient organisations/umbrella organisations/governments

1. Targeted google search for relevant organsations in Switzerland, Germany, EU and United States (16 April 2020 / 5 May 2020 and 6 May 2020)
    a. **Health insurances**

i. SVV – Schweizerischer Versicherungsverband (www.svv.ch) → no findings for "orphan drugs", "orphan drug", "seltene krankheiten"
ii. GKV (https://www.gkv-spitzenverband.de/) → 21 hits for "orphan drug"
iii. SGV – Schweiz. Gesellschaft der Vertrauens- und Versicherungsärzte → manual

- **Patient organisations**
  i. Eurordis (https://www.eurordis.org/de/positionspapiere)
  ii. Proraris
- **Governmental organisations**
  i. BAG → Nationale Strategie seltene Krankheiten
  ii. EUCERD / EC Expert Group on Rare Diseases
- **Industry**
  i. Vfa – die forschenden Pharmaunternehmen / https://www.vfa-bio.de/vb-de/aktuelle-themen/orphans
  ii. Vips
  iii. PharmaSuisse (www.pharmasuisse.org)
  iv. BPI
- **Institutions**
  i. IG Seltene Krankheiten
  ii. careum

2. Google and website search for relevant blogs / reports / policy papers etc. (5-6 May 2020)

- *"orphan drug versicherung" / "orphan drug reimbursement" / "orphan drug position paper" / "orphan drug policy paper" / "orphan drug recommendation"*

Additional file 1

Search summary

**Datenbank**	**Hits**
New Pubmed (medicine)	899
EMBASE (biomedicine)	4078
CINAHL (nursing)	407
WOS (all)	745
Philosopher's index (philosophy)	5
HeinOnline (law)	1750
Open grey	4
Google search / targeted position paper search	237
**Total**	**8121**
