# Supplementary material for: A systematic review of moral reasons on orphan drug reimbursement
Source: Orphanet J Rare Dis. 2021 Jun 30;16:292. doi: 10.1186/s13023-021-01925-y (PMC8247078; doi:10.1186/s13023-021-01925-y)
Supplement: Supplementary file 2 — Additional file 2. Article selection: excluded articles. Specifies how many articles were excluded on the full text screening stage for what reasons. [file 13023_2021_1925_MOESM2_ESM.pdf]

Additional File 2

Article selection: excluded articles

	No of articles	Percent of total
Excluded -> full text not available	97	8%
Excluded -> language not English or German	9	1%
Excluded -> format	137	11%
Excluded -> content	769	61%
Excluded -> double article	12	1%
Included	243	19%
**Total no of articles in full text screening**	1267	100%
