# Supplementary material for: A systematic review of moral reasons on orphan drug reimbursement
Source: Orphanet J Rare Dis. 2021 Jun 30;16:292. doi: 10.1186/s13023-021-01925-y (PMC8247078; doi:10.1186/s13023-021-01925-y)
Supplement: Supplementary file 3 — Additional file 3. Codebook. Includes variables and categories used for systematic data extraction. [file 13023_2021_1925_MOESM3_ESM.pdf]

Additional File 3

Codebook for data extraction

This codebook applies for data extraction in SPSS. Moral reasons are extracted inductively using MaxQDA.

1) Article number

2) Author list *

3) Title of article (including subtitles) *

4) Journal or book title *

5) Publication year *

6) Language
   1 English
   2 German

7) Article type
   1 Journal article
   2 Book chapter
   3 Dissertation
   4 Grey literature

8) Methodology
   1 Theoretical
   2 Qualitative (including case studies & mixed methods)
   3 Quantitative
   4 Comment/editorial
   5 Reviews of policies a.o.
   6 Systematic literatue reviews (incl. scoping reviews)

* extraction automated from citavi reference manager

7 Guideline / policy paper

8 Legal paper

9 Unclear / unknown

9) Discipline

Depending on content, then 1st author, then journal

1 Philosophy / bioethics (research)

2 Clinical medicine (research)

3 Health economics (research)

4 Health policy (research)

5 Law (research)

6 Social sciences (research)

7 Industry (stakeholder)

8 Patients (stakeholder)

10) Level of moral reasoning

1 Moral reasoning for/against special status was the main focus of the article

2 Moral reasoning was present extensively (more than one paragraph), but not the only focus

3 Moral reasoning was only marginal, mentioned in one paragraph or less

9 Unclear *(please state why in commentary)*

11) Country/countries the article is referring to (string)

State individual countries; if the article is not referring to any specific country, state "not specified"

12) Geographical region *(categories built and applied based on string data from variable 11)*

0 Not specified

1 Western Europe

2 Eastern Europe

3 Asia

4 United States

5 Canada

6 South America

7 Africa

8 Australia / New Zealand
9 International
99 Unclear/unknown

13) Scope of the article regarding OD
1 All medical products with an orphan designation
2 A group of drugs covering a specific disease (or group of disease, e.g. cancer oncology drugs)
3 One specific drug
4 Other
9 Unclear / unknown

14) Main conclusions of the study regarding orphan drug reimbursement*
1 Special status of OD: yes
2 Special status of OD: no
3 Special status of OD: conditionally
9 Unclear/unknown

15) Author's conflict of interest
1 Yes
2 No
9 Not available or applicable

16) Coder's commentary (string)
