# Supplementary material for: A systematic review of moral reasons on orphan drug reimbursement
Source: Orphanet J Rare Dis. 2021 Jun 30;16:292. doi: 10.1186/s13023-021-01925-y (PMC8247078; doi:10.1186/s13023-021-01925-y)
Supplement: Supplementary file 5 — Additional file 5. Specification of articles and moral reasons. Includes a table as a supplementary to Figure 3, specifies which articles contain what moral reasons. [file 13023_2021_1925_MOESM5_ESM.pdf]

Additional File 5

Specification of articles and moral reasons

Additional table 1: Moral reasons categories.

Moral reasons	n	References
**Maximise population health**	**118 (48.6%)**	
- OMPs do not maximise health of the population	63 (25.9%)	(1–63)
+ Individual needs should also be considered	6 (2.5%)	(40, 41, 64, 7, 65, 60)
- Covering OMPs means less money for common disorders	48 (19.8%)	(66, 4, 5, 61, 67, 68, 11, 69, 13, 70, 14, 71–73, 19, 62, 74–81, 29, 30, 82, 33, 83, 84, 36, 85, 86, 41, 87, 48, 50, 88, 54, 89, 57, 90–93, 60, 1, 7)
+ Low budget impact due to low prevalence	37 (15.2%)	(2, 94–96, 8, 10, 12, 97, 14, 15, 98, 72, 73, 18, 19, 21, 99–101, 33, 83, 84, 42, 45, 87, 102, 103, 88, 104, 105, 54, 106, 89, 107, 57, 92, 108)
- Taking all OMPs together, the budget impact is significant	27 (11.1%)	(109, 61, 69, 13, 73, 110, 111, 76, 77, 79, 112, 81, 113, 33, 83, 36, 41, 87, 47, 49, 114–116, 91, 117, 1, 118)
**Equality and equity**	**194 (79.8%)**	
- Special status of OMPs contradicts egalitarian principles	32 (13.2%)	(2, 119, 5, 120, 8, 67, 121, 69, 13, 18, 122, 76, 79, 29, 112, 101, 83, 38, 123, 43, 47, 50, 124, 125, 115, 54, 106, 55–57, 126, 1)
+ Special status of OMPs justified for reasons of equity	38 (15.6%)	(127, 5, 128, 129, 12, 97, 130, 122, 21, 76, 131, 33, 35, 85, 63, 123, 86, 41, 132, 50, 105, 54, 106, 90, 91, 133–135, 1)
+ RD patients should have the same access to treatment as others	78 (32.1%)	(136, 127, 3, 137, 138, 4, 139, 6, 109, 95, 96, 8–10, 121, 140, 69, 70, 97, 141, 142, 98, 143, 17, 144, 72, 145, 62, 110, 146, 74, 75, 147, 24, 27, 28, 112, 148, 81, 149, 30, 150–152, 34, 153, 84, 154, 155, 38, 39, 123, 156, 40, 64, 132, 87, 46, 157, 158, 47, 48, 159, 49, 160, 50, 65, 161–163, 124, 164, 56, 57, 126, 165, 58, 166)
+ All should have equal opportunities to good health	30 (12.3%)	(167, 61, 13, 97, 168, 73, 18, 122, 62, 145, 74, 147, 23, 78, 112, 33, 35, 36, 59, 37, 41, 64, 50, 162, 106, 57, 116, 126, 93, 1)
+ Prioritize OMPs to aim for equal health outcomes	6 (2.5%)	(13, 145, 74, 38, 156, 1)
+ Everybody has same right to a decent minimum standard of care	14 (5.8%)	(13, 15, 168, 18, 19, 74, 23, 30, 83, 84, 59, 41, 50, 49)
+ Prioritise those who are worse off	24 (9.9%)	(4, 7, 13, 70, 168, 122, 74, 77, 78, 36, 38, 39, 56, 169, 1, 170, 171, 12, 91, 37, 41, 42, 132, 48)
+ Prioritize OMPs because/if they treat severe rare diseases	95 (39.1%)	(172, 167, 8, 173, 174, 10, 175, 12, 98, 18, 21, 176, 76, 22, 23, 99, 177–179, 26, 112, 149, 101, 150, 83, 84, 36, 59, 86, 156, 180, 132, 87, 181, 47, 182, 48, 164, 88, 52, 104, 56, 126, 91, 92, 134, 165, 1, 127, 183, 184, 19, 62, 185, 186, 3, 138, 4, 187, 6, 120, 109, 61, 9, 11, 15, 71, 17, 73, 188, 20, 189, 75, 24, 78, 55, 148, 81, 190, 30, 57, 191, 37, 38, 40–42, 93, 43, 46, 192, 58, 49, 193)

Additional File 5

+ Prioritize RD patients with unmet needs	29 (11.9%)	(2, 172, 68, 98, 176, 99, 24, 177, 179, 194, 195, 150, 196, 83, 36, 85, 156, 43, 45, 197, 182, 48, 198, 161, 105, 71, 56, 91, 58)
+ Prioritize OMPs when no alternative treatment is available	68 (28%)	(3, 138, 172, 120, 109, 128, 173, 174, 10, 175, 121, 11, 12, 97, 98, 17, 199, 188, 122, 111, 21, 146, 176, 23, 99, 177, 186, 27, 80, 29, 112, 81, 149, 31, 150, 191, 83–85, 38, 156, 180, 200, 132, 43, 45, 201, 197, 47, 202, 182, 48, 184, 193, 65, 187, 52, 53, 104, 189, 56, 91, 185, 92, 133, 117, 134, 165)
+ Special status because OMPs are too expensive for self-payment	22 (9.1%)	(173–175, 12, 17, 178, 28, 183, 155, 85, 132, 87, 203, 182, 102, 184, 204, 205, 92, 117, 134, 108)
+ RD patients are disadvantaged due to rarity	17 (7%)	(136, 66, 9, 206, 122, 145, 147, 77, 207, 208, 151, 63, 86, 41, 184, 1, 57)
**Personal responsibility**	**9 (3.7%)**	
+ RD patients are sick out of bad luck	4 (1.6%)	(13, 149, 106, 57)
Society values individual lifestyle choices	5 (2.1%)	(57, 41, 42, 182, 93)
**Rule of Rescue**	**62 (25.5%)**	
+ Moral intuition to rescue the identifiable no matter cost	48 (19.8%)	(209, 94, 4, 210, 172, 109, 8, 121, 13–15, 168, 73, 18–20, 147, 77, 78, 207, 25, 27, 30, 31, 191, 34, 83, 153, 211, 212, 85, 38, 39, 123, 41, 42, 7, 43, 45, 46, 159, 50, 51, 107, 52, 92, 60, 1)
- Concept of the Rule of Rescue is inappropriate	20 (8.2%)	(61, 121, 12, 168, 20, 76, 99, 77, 78, 25, 28, 80, 208, 113, 33, 43, 7, 88, 52, 1)
- The aspect of identifiability is not acceptable	21 (8.6%)	(61, 168, 74, 77, 78, 207, 80, 29, 30, 33, 83, 40, 41, 1, 50, 7, 51, 88, 92, 60, 49)
**Duty**	**63 (25.9)**	
+ Society has a duty to provide effective treatment if available'	28 (11.5%)	(127, 213, 214, 139, 215, 96, 168, 145, 76, 23, 84, 35, 36, 59, 216, 40, 46, 192, 48, 160, 217, 50, 161, 105, 106, 57, 91, 93)
+ OMP reimbursement conforms to principle of social solidarity	21 (8.6%)	(137, 218, 95, 173, 206, 14, 130, 76, 78, 37, 180, 219, 41, 132, 182, 50, 105, 189, 57)
+ Receiving appropriate treatment is a human right	20 (8.2%)	(94, 10, 12, 70, 97, 130, 62, 113, 150, 33, 83, 38, 219, 41, 44, 46, 220, 221, 117, 222)
+ Doctor's duty to provide treatment in the interest of patients	8 (3.3%)	(210, 9, 62, 20, 76, 27, 46, 106)
**Rarity**	**77 (31.7%)**	
+ Rarity in itself is a factor that warrants special status	23 (9.5%)	(127, 61, 96, 11, 72, 188, 78, 195, 180, 41, 42, 132, 87, 223, 182, 224, 220, 125, 104, 56, 221, 91, 1)
+ Societal preference for special status for rare diseases	17 (7%)	(4, 12, 14, 18, 188, 122, 145, 101, 83, 84, 37, 219, 225, 189, 56, 1)
- Rarity in itself does not justify special status	19 (7.8%)	(9, 69, 122, 74, 99, 29, 226, 83, 154, 86, 182, 1, 198, 193, 89, 118, 126, 221)
- No societal preference for rarity alone	42 (17.3%)	(127, 227, 138, 4, 5, 172, 61, 171, 8–10, 121, 68, 11, 12, 62, 145, 20, 179, 186, 81, 80, 228, 30, 101, 191, 83, 154, 37, 85, 41, 132, 43, 47, 182, 48, 198, 49, 187, 56, 229, 1)

Additional File 5

**Appropriateness of cost-effectiveness criteria**	**103 (42.4%)**	
- OMPs can & should meet same cost-effec criteria as other drugs	19 (7.8%)	(4, 120, 96, 11, 73, 19, 74, 80, 29, 112, 83, 59, 41, 47, 51, 103, 104, 54, 166)
+ OMPs unlikely to meet traditional cost-effec criteria	52 (21.4%)	(127, 2, 227, 4, 172, 174, 173, 175, 9, 129, 121, 11, 97, 168, 122, 110, 21, 230, 231, 24, 78, 232, 26, 194, 195, 29, 31, 32, 113, 150, 35, 85, 123, 180, 219, 40, 41, 7, 43, 87, 182, 198, 114, 125, 89, 56, 126, 91, 233, 133, 58, 1)
+ Standard economic assessment inappropriate for OMPs	54 (22.2%)	(127, 218, 227, 4, 61, 8, 10–12, 69, 70, 98, 72, 73, 19, 122, 145, 111, 21, 146, 230, 76, 78, 177, 26, 234, 190, 101, 150, 83, 235, 37, 123, 40, 42, 236, 64, 43, 182, 48, 237, 7, 114, 88, 164, 115, 238, 104, 54, 89, 107, 185, 117, 58)
+ Reimburse if certain standards are met	25 (10.3%)	(120, 61, 67, 121, 13, 97, 98, 73, 111, 21, 146, 176, 78, 191, 83, 219, 40, 64, 182, 198, 51, 105, 89, 56, 57)
**Treatment benefit**	**76 (31.3%)**	
+ Benefits of OMPs justify special status	48 (19.8%)	(94, 138, 172, 167, 96, 9–13, 97, 98, 73, 188, 122, 62, 20, 176, 76, 99, 78, 177, 100, 239, 186, 81, 190, 30, 101, 150, 83, 84, 235, 85, 123, 156, 40, 132, 47, 182, 48, 224, 105, 89, 189, 56, 57, 240)
+ OMPs can lower social/economic burden of society	25 (10.3%)	(139, 167, 70, 62, 110, 21, 146, 26, 100, 239, 190, 83, 84, 63, 236, 87, 48, 184, 65, 224, 189, 91, 185, 92, 240)
OMP should have proven effectiveness & safety	26 (10.7%)	(2, 215, 97, 206, 15, 98, 111, 146, 176, 232, 29, 112, 83, 35, 85, 40, 181, 47, 182, 48, 7, 163, 105, 57, 165, 166)
**R&D investments**	**49 (20.2%)**	
+ Reimbursing OMPs incentivises more R&D in the future	38 (15.6%)	(96, 171, 9, 140, 97, 72, 18, 21, 176, 179, 194, 239, 27, 28, 112, 196, 83, 84, 216, 43, 87, 223, 184, 193, 217, 65, 224, 54, 106, 89, 189, 107, 56, 241, 57, 91, 117, 165)
+ Duty to reimburse OMPs because so much investment into R&D	19 (7.8%)	(213, 218, 96, 11, 12, 97, 242, 243, 112, 81, 30, 208, 83, 84, 132, 223, 224, 57, 125)
**Total no of documents**	243 (100%)	

Notes: n = number of documents this reason has been mentioned. + = Reason in favor of special status of OMPs. – = Reason against special status of OMPs.

Included articles

1. Wiss J. Healthcare Priority Setting and Rare Diseases: What Matters When Reimbursing Orphan Drugs. Linköping University Electronic Press; 2017.

2. Babar Z-U-D, Francis S. Identifying priority medicines policy issues for New Zealand: a general inductive study. BMJ OPEN 2014; 4(5).

3. Bilkey GA, Burns BL, Coles EP, Mahede T, Baynam G, Nowak KJ. Optimizing Precision Medicine for Public Health. FRONTIERS IN PUBLIC HEALTH 2019; 7:42.

4. Brenna E, Polistena B, Spandonaro F. The implementation of health technology assessment principles in public decisions concerning orphan drugs. EUROPEAN JOURNAL OF CLINICAL PHARMACOLOGY 2020.

5. Burls A, Austin D, Moore D. Commissioning for rare diseases: view from the frontline. BMJ 2005; 331(7523):1019–21.

6. Clarke JTR. The Price of Care Versus the Cost of Caring. In: Elstein D, Altarescu G, Beck M, editors. FABRY DISEASE; 2010. p. 489–97.

7. da Silva EN, Vieira Sousa TR. Economic evaluation in the context of rare diseases: is it possible? CADERNOS DE SAUDE PUBLICA 2015; 31(3):496–506.

8. Dear JW, Lilitkarntakul P, Webb DJ. Are rare diseases still orphans or happily adopted? The challenges of developing and using orphan medicinal products. BRITISH JOURNAL OF CLINICAL PHARMACOLOGY 2006; 62(3):264–71.

9. Desser AS. Prioritizing treatment of rare diseases: a survey of preferences of Norwegian doctors. Soc Sci Med 2013; 94:56–62.

10. Desser AS, Gyrd-Hansen D, Olsen JA, Grepperud S, Kristiansen IS. Societal views on orphan drugs: cross sectional survey of Norwegians aged 40 to 67. BRITISH MEDICAL JOURNAL 2010; 341.

11. Drummond M, Towse A. Orphan drugs policies: a suitable case for treatment. EUROPEAN JOURNAL OF HEALTH ECONOMICS 2014; 15(4):335–40.

12. Drummond M, Wilson DA, Kanavos P, Ubel P, Rovira J. Assessing the economic challenges posed by orphan drugs. INTERNATIONAL JOURNAL OF TECHNOLOGY ASSESSMENT IN HEALTH CARE 2007; 23(1):36–42.

13. Ehni H-J. Expensive cancer drugs and just health care. Best Pract Res Clin Gastroenterol 2014; 28(2):327–37.

14. Feldman B.M., Berger K., Bohn R., Carcao M., Fischer K., Gringeri A. et al. Haemophilia prophylaxis: How can we justify the costs? HAEMOPHILIA 2012; 18(5):680–4. Available from: URL: http://www.embase.com/search/results?subaction=viewrecord&from=export&id=L51967846.

15. Fishman J.C., Skrepnek G.H. Pharmacoeconomic analyses of treatments for rare disease. Pharmaceuticals Policy and Law 2012; 14(1):51–67. Available from: URL: http://www.embase.com/search/results?subaction=viewrecord&from=export&id=L364245786.

16. Gilabert-Perramon A., Torrent-Farnell J, Catalan A., Prat A., Fontanet M., Puig-Peiró R. et al. Drug evaluation and decision making in catalonia: Development and validation of a methodological framework based on multi-criteria decision analysis (MCDA) for orphan drugs. INTERNATIONAL JOURNAL OF TECHNOLOGY ASSESSMENT IN HEALTH CARE 2017; 33(1):111–20. Available from: URL: http://www.embase.com/search/results?subaction=viewrecord&from=export&id=L615666039.

17. Gonzato O. The new risk-sharing paradigm in rare cancers: Patient perspective. Journal of Cancer Policy 2017; 12:36–42. Available from: URL: http://www.embase.com/search/results?subaction=viewrecord&from=export&id=L614830961.

18. Hughes D, Tunnage B, Yeo ST. Drugs for exceptionally rare diseases: do they deserve special status for funding? QJM-AN INTERNATIONAL JOURNAL OF MEDICINE 2005; 98(11):829–36.

19. Hughes D. Rationing of drugs for rare diseases. PHARMACOECONOMICS 2006; 24(4):315–6.

20. Isaacs D. Ethical dilemmas about orphan drugs for orphan diseases. JOURNAL OF PAEDIATRICS AND CHILD HEALTH 2014; 50(4):249–50. Available from: URL: http://www.embase.com/search/results?subaction=viewrecord&from=export&id=L372806321.

21. Iskrov G, Miteva-Katrandzhieva T, Stefanov R. Health Technology Assessment and Appraisal of Therapies for Rare Diseases. In: DeLaPaz MP, Taruscio D, Groft SC, editors. RARE DISEASES EPIDEMIOLOGY: UPDATE AND OVERVIEW, 2ND EDITION. GEWERBESTRASSE 11, CHAM, CH-6330, SWITZERLAND: SPRINGER INTERNATIONAL PUBLISHING AG; 2017. p. 221–31 (Advances in Experimental Medicine and Biology).

22. Kesselman I, Elstein D, Israeli A, Chertkoff R, Zimran A. National health budgets for expensive orphan drugs: Gaucher disease in Israel as a model. BLOOD CELLS MOLECULES AND DISEASES 2006; 37(1):46–9.

23. Kinney J. Health disparities: Exploring the ethics of orphan drugs. AM J HEALTH SYST PHARM AJHP 2014; 71(9):692–3. Available from: URL: http://search.ebscohost.com/login.aspx?direct=true&db=cin20&AN=103939421&site=ehost-live.

24. Korchagina D, Jaroslawski S, Jadot G, Toumi M. Orphan Drugs in Oncology. Recent Results Cancer Res 2019; 213:109–42.

25. London AJ. How should we model rare disease allocation decisions? Hastings Cent Rep 2012; 42(1):3.

26. López-Bastida J, Oliva-Moreno J. Cost of illness and economic evaluation in rare diseases. In: DeLaPaz MP, Taruscio D, Groft SC, editors. RARE DISEASES EPIDEMIOLOGY: UPDATE AND OVERVIEW, 2ND EDITION. GEWERBESTRASSE 11, CHAM, CH-6330, SWITZERLAND: SPRINGER INTERNATIONAL PUBLISHING AG; 2017. p. 273–82 (Advances in Experimental Medicine and Biology).

27. Mavroudis C, Jacobs JP. The elephant in the room: ethical issues associated with rare and expensive medical conditions. Cardiol Young 2015; 25(8):1621–5.

28. McCabe C. Balancing economic, ethical and equity concerns in orphan drugs and rare diseases. Eur J Hosp Pharm Pract 2010; 16(4):22–5.

29. McCabe C, Claxton K, Tsuchiya A. Orphan drugs and the NHS: should we value rarity? BMJ 2005; 331(7523):1016–9.

30. Mentzakis E, Stefanowska P, Hurley J. A discrete choice experiment investigating preferences for funding drugs used to treat orphan diseases: an exploratory study. Health Econ Policy Law 2011; 6(3):405–33.

31. Mincarone P, Leo CG, Sabina S, Sarria-Santamera A, Taruscio D, Guillermo Serrano-Aguilar P et al. Reimbursed Price of Orphan Drugs: Current Strategies and Potential Improvements. PUBLIC HEALTH GENOMICS 2017; 20(1):1–8.

32. Monahan AB. Fairness versus Welfare in Health Insurance Content Regulation. U. Ill. L. Rev. 2012; 2012:139. Available from: URL: https://heinonline.org/HOL/Page?public=true&handle=hein.journals/unilllr2012&div=7&start_page=139&collection=journals&set_as_cursor=483&men_tab=srchresults.

33. Ollendorf DA, Chapman RH, Pearson SD. Evaluating and Valuing Drugs for Rare Conditions: No Easy Answers. VALUE IN HEALTH 2018; 21(5):547–52.

34. Panju AH, Bell CM. Policy alternatives for treatments for rare diseases. CMAJ 2010; 182(17):E787-92.

35. Picavet E, Cassiman D, Pinxten W, Simoens S. Ethical, legal and social implications of rare diseases and orphan drugs in Europe: Meeting report of a Brocher symposium. Expert Review of Pharmacoeconomics and Outcomes Research 2013; 13(5):571–3. Available from: URL: http://www.embase.com/search/results?subaction=viewrecord&from=export&id=L370115405.

36. Pinxten W, Denier Y, Dooms M, Cassiman J-J, Dierickx K. A fair share for the orphans: ethical guidelines for a fair distribution of resources within the bounds of the 10-year-old European Orphan Drug Regulation. Journal of Medical Ethics 2012; 38(3):148–53.

37. Richardson J, Iezzi A, Chen G, Maxwell A. Communal Sharing and the Provision of Low-Volume High-Cost Health Services: Results of a Survey. Pharmacoecon Open 2017; 1(1):13–23.

38. Rodriguez-Monguio R, Spargo T, Seoane-Vazquez E. Ethical imperatives of timely access to orphan drugs: is possible to reconcile economic incentives and patients' health needs? ORPHANET JOURNAL OF RARE DISEASES 2017; 12(1):1.

39. Roll K, Stargardt T, Schreyögg J. Authorization and Reimbursement of Orphan Drugs in an International Comparison. GESUNDHEITSWESEN 2011; 73(8-9):504–14.

40. Schlander M, Beck M. Expensive drugs for rare disorders: to treat or not to treat? The case of enzyme replacement therapy for mucopolysaccharidosis VI. CURRENT MEDICAL RESEARCH AND OPINION 2009; 25(5):1285–93.

41. Schlander M, Garattini S, Holm S, Kolominsky-Rabas P, Nord E, Persson U et al. Incremental cost per quality-adjusted life year gained? The need for alternative methods to evaluate medical interventions for ultra-rare disorders. JOURNAL OF COMPARATIVE EFFECTIVENESS RESEARCH 2014; 3(4):399–422.

42. Schlander M, Garattini S, Kolominsky-Rabas P, Nord E, Persson U, Postma MJ et al. Determining the value of medical technologies to treat ultra-rare disorders: a consensus statement. J Mark Access Health Policy 2016; 4.

43. Simoens S, Cassiman D, Dooms M, Picavet E. Orphan Drugs for Rare Diseases Is it Time to Revisit Their Special Market Access Status? DRUGS 2012; 72(11):1437–43.

44. Simoens S, Picavet E, Dooms M, Cassiman D, Morel T. Cost-effectiveness assessment of orphan drugs: a scientific and political conundrum. APPLIED HEALTH ECONOMICS AND HEALTH POLICY 2013; 11(1):1–3.

45. Simon F. Market access for biopharmaceuticals: new challenges. Health Aff (Millwood) 2006; 25(5):1363–70.

46. Taylor C, Jan S, Thompson K. Funding therapies for rare diseases: an ethical dilemma with a potential solution. AUSTRALIAN HEALTH REVIEW 2018; 42(1):117–9.

47. van Egmond-Froehlich A, Schmitt K, AG Schwerpunktsetzung Subspezi. Public guidance and price limitation for orphan drugs. Fair distribution and right to treatment of rare diseases. MONATSSCHRIFT KINDERHEILKUNDE 2018; 166(9):785–97.

48. Wagner M, Khoury H, Willet J., Rindress D, Goetghebeur M. Can the EVIDEM Framework Tackle Issues Raised by Evaluating Treatments for Rare Diseases: Analysis of Issues and Policies, and Context-Specific Adaptation. PHARMACOECONOMICS 2016; 34(3):285–301. Available from: URL: http://www.embase.com/search/results?subaction=viewrecord&from=export&id=L606858403.

49. Wiss J, Levin L-A, Andersson D, Tinghoeg G. Prioritizing Rare Diseases: Psychological Effects Influencing Medical Decision Making. MEDICAL DECISION MAKING 2017; 37(5):567–76.

50. Zelei T, Molnar MJ, Szegedi M, Kalo Z. Systematic review on the evaluation criteria of orphan medicines in Central and Eastern European countries. ORPHANET JOURNAL OF RARE DISEASES 2016; 11.

51. Kaebnick G. Two dreams. Hastings Cent Rep 2012; 42(1):2.

52. Cookson R, McCabe C, Tsuchiya A. Public healthcare resource allocation and the Rule of Rescue. J MED ETHICS 2008; 34(7):540–4.

53. Devlin N, Parkin D. Does NICE have a cost-effectiveness threshold and what other factors influence its decisions? A binary choice analysis. HEALTH ECONOMICS 2004; 13(5):437–52.

54. Hollis A. Drugs for rare diseases: paying for innovation. In: Beach CM, editor. Health services restructuring in Canada: New evidence and new directions. Montreal: McGill-Queen's Univ. Press; 2006. p. 155–77.

55. Leget C, Hoedemaekers R. Teaching medical students about fair distribution of healthcare resources. Journal of Medical Ethics: The Journal of the Institute of Medical Ethics 2007; 33(12):737–41.

56. Medic G, Korchagina D, Young KE, Toumi M, Postma MJ, Wille M et al. Do payers value rarity? An analysis of the relationship between disease rarity and orphan drug prices in Europe. J Mark Access Health Policy 2017; 5(1):1299665.

57. National Institute for Clinical Excellence. Citizens Council report: Ultra Orphan drugs. London; 2004 [cited 2020 Nov 18]. Available from: URL: https://www.ncbi.nlm.nih.gov/books/NBK401721/pdf/Bookshelf_NBK401721.pdf.

58. Wild C, Hintringer K, Nachtnebel A. Orphan drugs in oncology. In: Walter E, editor. Regulatory and Economic Aspects in Oncology. Cham: Springer International Publishing; 2019. p. 223–32 (vol. 213).

59. Rai AK. Pharmacogenetic interventions, orphan drugs, and distributive justice: the role of cost-benefit analysis. Soc Philos Policy 2002; 19(2):246–70.

60. Sheehan M. Resources and the Rule of Rescue. J Appl Philos 2007; 24(4):352–66.

61. Coyle D, Cheung MC, Evans G. Opportunity Cost of Funding Drugs for Rare Diseases: The Cost-Effectiveness of Eculizumab in Paroxysmal Nocturnal Hemoglobinuria. MED DECIS MAKING 2014; 34(8):1016–29. Available from: URL: http://search.ebscohost.com/login.aspx?direct=true&db=cin20&AN=98946387&site=ehost-live.

62. Hyry HI, Roos JCP, Manuel J, Cox TM. The legal imperative for treating rare disorders. ORPHANET JOURNAL OF RARE DISEASES 2013; 8:135.

63. Roscamp JA, D'Cruz DP. The funding lottery for potentially life-threatening rare diseases It's not fair, my disease is rare. RHEUMATOLOGY 2018; 57(1):1–2.

64. Sheehan M. Orphan drugs and the NHS: fairness in health care entails more than cost effectiveness…McCabe C, Claxton K, Tsuchiya A. Orphan drugs and the NHS: should we value rarity? BMJ 2005;331:1016-9. (29 October). BMJ 2005; 331(7525):1144–5. Available from: URL: http://search.ebscohost.com/login.aspx?direct=true&db=cin20&AN=106368373&site=ehost-live.

65. BPI-Positionspapier Orphan Drugs: Compassionate Use - Wirtschaftliche Anreize - Verordnungsfähigkeit zur Lasten der GKV. Berlin; 2008.

66. Blankart CR, Stargardt T, Schreyögg J. Availability of and access to orphan drugs: an international comparison of pharmaceutical treatments for pulmonary arterial hypertension, Fabry disease, hereditary angioedema and chronic myeloid leukaemia. PHARMACOECONOMICS 2011; 29(1):63–82.

67. Denis A, Mergaert L, Fostier C, Cleemput I., Simoens S. Budget impact analysis of orphan drugs in Belgium: Estimates from 2008 to 2013. Journal of Medical Economics 2010; 13(2):295–301. Available from: URL: http://www.embase.com/search/results?subaction=viewrecord&from=export&id=L358981528.

68. Dragojlovic N, Rizzardo S, Bansback N, Mitton C, Marra C, Lynd LD. Challenges in Measuring the Societal Value of Orphan Drugs: Insights from a Canadian Stated Preference Survey. PATIENT-PATIENT CENTERED OUTCOMES RESEARCH 2015; 8(1):93–101.

69. Dupont AG, van Wilder PB. Access to orphan drugs despite poor quality of clinical evidence. BRITISH JOURNAL OF CLINICAL PHARMACOLOGY 2011; 71(4):488–96.

70. Encina G, Castillo-Laborde C, Lecaros JA, Dubois-Camacho K, Calderon JF, Aguilera X et al. Rare diseases in Chile: challenges and recommendations in universal health coverage context. ORPHANET JOURNAL OF RARE DISEASES 2019; 14(1).

71. Goetghebeur M, Wagner M. Identifying Value(s): A Reflection on the Ethical Aspects of MCDA in Healthcare Decisionmaking. In: Marsh K, Goetghebeur M, Thokala P, Baltussen R, editors. Multi-criteria decision analysis to support healthcare decisions. Springer; 2017. p. 29–46.

72. Hasselmann O. Sind hohe Preise für "Orphan Drugs" ethisch zu rechtfertigen? Epileptologie 2013; (30):72–8. Available from: URL: https://www.epi.ch/wp-content/uploads/Artikel-Hasselmann_1_13.pdf.

73. Houlton S. Orphan medicines: The high cost of hope. Prescriber 2018; 29(1):23–7. Available from: URL: http://www.embase.com/search/results?subaction=viewrecord&from=export&id=L620325181.

74. Juth N. For the Sake of Justice: Should We Prioritize Rare Diseases? Health Care Anal 2017; 25(1):1–20. Available from: URL: http://www.embase.com/search/results?subaction=viewrecord&from=export&id=L618378960.

75. Kanavos P, Nicod E. What is wrong with orphan drug policies? Suggestions for ways forward. Value Health 2012; 15(8):1182–4.

76. Karpman D, Hoglund P. Orphan drug policies and use in pediatric nephrology. PEDIATRIC NEPHROLOGY 2017; 32(1):1–6.

77. Kling S. Allocating treatment for rare allergic diseases - The rule of rescue. Current Allergy and Clinical Immunology 2013; 26(2):94–6. Available from: URL: http://www.embase.com/search/results?subaction=viewrecord&from=export&id=L369166268.

78. Largent EA, Pearson SD. Which orphans will find a home? The rule of rescue in resource allocation for rare diseases. Hastings Cent Rep 2012; 42(1):27–34.

79. Laupacis A. Economic evaluations in the Canadian common drug review. PHARMACOECONOMICS 2006; 24(11, SI):1157–62.

80. McCabe C, Tsuchiya A, Claxton K, Raftery J. Orphan drugs revisited. QJM 2006; 99(5):341–5. Available from: URL: http://www.embase.com/search/results?subaction=viewrecord&from=export&id=L43651026.

81. McCabe C, Tsuchiya A, Claxton K, Raftery J. Assessing the economic challenges posed by orphan drugs: a comment on Drummond et al. INTERNATIONAL JOURNAL OF TECHNOLOGY ASSESSMENT IN HEALTH CARE 2007; 23(3):397-401; author reply 401-4.

82. Newdick C. Accountability for Rationing - Theory into Practice. J.L. Med. & Ethics 2005; 33:660. Available from: URL: https://heinonline.org/HOL/Page?public=true&handle=hein.journals/medeth33&div=77&start_page=660&collection=journals&set_as_cursor=51&men_tab=srchresults.

83. Paulden M, Stafinski T, Menon D, McCabe C. Value-Based Reimbursement Decisions for Orphan Drugs: A Scoping Review and Decision Framework. PHARMACOECONOMICS 2015; 33(3):255–69.

84. Picavet E, Dooms M, Cassiman D, Simoens S. Orphan Drugs for Rare Diseases: Grounds for Special Status. DRUG DEVELOPMENT RESEARCH 2012; 73(3):115–9.

85. Rizzardo S, Bansback N, Dragojlovic N, Douglas C, Li KH, Mitton C et al. Evaluating Canadians' Values for Drug Coverage Decision Making. VALUE HEALTH 2019; 22(3):362–9. Available from: URL: http://search.ebscohost.com/login.aspx?direct=true&db=cin20&AN=135351895&site=ehost-live.

86. Sandman L, Gustavsson E. The (Ir)relevance of Group Size in Health Care Priority Setting: A Reply to Juth. HEALTH CARE ANAL 2017; 25(1):21–33. Available from: URL: http://search.ebscohost.com/login.aspx?direct=true&db=cin20&AN=121302247&site=ehost-live.

87. Tambuyzer E. Rare diseases, orphan drugs and their regulation: Questions and misconceptions. Nat Rev Drug Discov 2010; 9(12):921–9. Available from: URL: http://www.embase.com/search/results?subaction=viewrecord&from=export&id=L51144273.

88. Clarke JTR, Amato D, Deber RB. Managing public payment for high-cost, high-benefit treatment: enzyme replacement therapy for Gaucher's disease in Ontario. CMAJ 2001; 165(5):595–6.

89. Iskrov G, Stefanov R. Post-marketing access to orphan drugs: a critical analysis of health technology assessment and reimbursement decision-making considerations. ODRR 2014:1.

90. Paulden M. Recent amendments to NICE's value-based assessment of health technologies: implicitly inequitable? Expert Review of Pharmacoeconomics and Outcomes Research 2017; 17(3):239–42.

91. Picavet E, Cassiman D, Simoens S. Do ultra-orphan medicinal products warrant ultra-high prices? A review. ODRR 2013:23.

92. Rosselli D, Rueda J-D, Solano M. Ethical and economic considerations of rare diseases in ethnic minorities: the case of mucopolysaccharidosis VI in Colombia. J MED ETHICS 2012; 38(11):699–700.

93. Schlander M, Holm S, Nord E, Richardson J, Garattini S, Kolominsky-Rabas P et al. Towards Social Cost Value Analysis: The Need for New Approaches for Evaluating Drugs for Ultra-Rare Diseases (URDs); 2016. Position Paper No 31 [cited 2020 Nov 19]. Available from: URL: http://www.innoval-hc.com/discussion-papers.html?file=files/publications/disc_papers/2015/IV-DP-31-Towards-SCVA-for-URDs-Oct-2015-March-v2-2016.pdf.

94. Banon Hernandez AM, Solves Almela JA. THE DEBATE ON RARE DISEASES A LOOK AT MEDIA RESPONSE. METODE SCIENCE STUDIES JOURNAL 2016; (6):209–13.

95. Czech M, Baran-Kooiker A, Atikeler K, Demirtshyan M, Gaitova K, Holownia-Voloskova M et al. A Review of Rare Disease Policies and Orphan Drug Reimbursement Systems in 12 Eurasian Countries. FRONTIERS IN PUBLIC HEALTH 2020; 7.

96. Davies JE, Neidle S, Taylor DG. Developing and paying for medicines for orphan indications in oncology: utilitarian regulation vs equitable care? BRITISH JOURNAL OF CANCER 2012; 106(1):14–7.

97. Faeh A. A Just Distribution of Health Care in the Case of Orphan Medicinal Products: Aligning the Interests of European Economic Integration and National Welfare Policy. Eur. J. Soc. Sec. (March) 2012; 14:0. Available from: URL: https://heinonline.org/HOL/Page?public=true&handle=hein.journals/eujsocse14&div=6&start_page=21&collection=journals&set_as_cursor=0&men_tab=srchresults.

98. Goetghebeur M, Wagner M, Samaha D, O'Neil W., Badgley D., Castro-Jaramillo H. et al. Exploring values of health technology assessment agencies using reflective multicriteria and rare disease case. INTERNATIONAL JOURNAL OF TECHNOLOGY ASSESSMENT IN HEALTH CARE 2017; 33(4):504–20. Available from: URL: http://www.embase.com/search/results?subaction=viewrecord&from=export&id=L618796957.

99. Kleinhout-Vliek T, Bont A de, Boer B. The bare necessities? A realist review of necessity argumentations used in health care coverage decisions. HEALTH POLICY 2017; 121(7):731–44.

100. Lumry WR. Pharmacoeconomics of Orphan Disease Treatment with a Focus on Hereditary Angioedema. Immunol Allergy Clin North Am 2017; 37(3):617–28.

101. Moberly T. Rationing and access to orphan drugs. Pharmaceutical Journal 2005; 275(7374):569–70. Available from: URL: https://www.pharmaceutical-journal.com/rationing-and-access-to-orphan-drugs/20015978.article?firstPass=false.

102. Weismann MF, Jorge I. The Regulatory Vision of Universal Healthcare in the United States: Strategic, Economic, and Moral Decision-Making. U. Pa. J. Bus. L. 2018; 21:647. Available from: URL: https://heinonline.org/HOL/Page?public=true&handle=hein.journals/upjlel21&div=20&start_page=647&collection=journals&set_as_cursor=309&men_tab=srchresults.

103. Erweitertes vips-Positionspapier zu einer Orphan-Drug-Strategie in der Schweiz: Handlungsfelder und Lösungsvorschläge; December 2011 [cited 5.5.20]. Available from: URL: https://docplayer.org/67943137-Erweitertes-vips-positionspapier-zu-einer-orphan-drug-strategie-in-der-schweiz-handlungsfelder-und-loesungsvorschlaege.html.

104. Drummond M. Challenges in the economic evaluation of orphan drugs. Eurohealth 2008; 14(2):16–7.

105. Gershon G. A report of the Ontario citizens' council considerations for funding drugs for rare diseases. Toronto, Ontario: Ontario Ministry of Health; 2010 [cited 2020 Nov 17]. Available from: URL: http://www.health.gov.on.ca/en/public/programs/drugs/councils/docs/report_201003.pdf.

106. Hunter D, Wilson J. Hyper-expensive treatments: Background paper. London; 2011 [cited 2020 Nov 17]. Available from: URL: https://discovery.ucl.ac.uk/id/eprint/1325654/1/Hyper_expensive_treatments_background_paper.pdf.

107. Kessabi S, Abreu Lourenco R de, Wonder M. Rescuing patients from the rule of efficiency: a need to debate the 'rule of rescue'. PHARMACOECONOMICS 2003; 21(9):681.

108. Reinhardt U. Probing Our Moral Values in Health Care: The Pricing of Specialty Drugs. JAMA - Journal of the American Medical Association 2015; 314(10):981–2.

109. Cote A, Keating B. What Is Wrong with Orphan Drug Policies? VALUE IN HEALTH 2012; 15(8):1185–91.

110. Iskrov G, Dermendzhiev S, Miteva-Katrandzhieva T, Stefanov R. Health Economic Data in Reimbursement of New Medical Technologies: Importance of the Socio-Economic Burden as a Decision-Making Criterion. FRONTIERS IN PHARMACOLOGY 2016; 7.

111. Iskrov G, Miteva-Katrandzhieva T, Stefanov R. Multi-criteria Decision analysis for assessment and appraisal of Orphan Drugs. FRONTIERS IN PUBLIC HEALTH 2016; 4.

112. McCabe C, Edlin R, Round J. Economic considerations in the provision of treatments for rare diseases. In: DeLaPaz MP, Taruscio D, Groft SC, editors. RARE DISEASES EPIDEMIOLOGY: UPDATE AND OVERVIEW, 2ND EDITION. GEWERBESTRASSE 11, CHAM, CH-6330, SWITZERLAND: SPRINGER INTERNATIONAL PUBLISHING AG; 2017. p. 211–22 (Advances in Experimental Medicine and Biology).

113. Moore D, Ries M, Forget EL, Schiffmann R. Enzyme replacement therapy in orphan and ultra-orphan diseases - The limitations of standard economic metrics as exemplified by Fabry-Anderson disease. PHARMACOECONOMICS 2007; 25(3):201–8.

114. Carrera P, IJzerman MJ. Are current ICER thresholds outdated? Valuing medicines in the era of personalized healthcare. EXPERT REVIEW OF PHARMACOECONOMICS & OUTCOMES RESEARCH 2016; 16(4):435–7.

115. Denis A, Simoens S, Fostier C, Mergaert L, Cleemput I. Policies for Orphan Diseases and Orphan Drugs; 2009 [cited 2020 Nov 16]. Available from: URL: https://ec.europa.eu/health/ph_threats/non_com/docs/policies_orphan_en.pdf.

116. Niezen MGH, Bont A de, Busschbach JJV, Cohen JP, Stolk EA. Finding legitimacy for the role of budget impact in drug reimbursement decisions. INTERNATIONAL JOURNAL OF TECHNOLOGY ASSESSMENT IN HEALTH CARE 2009; 25(1):49–55.

117. Simoens S, Dooms M. Market access of orphan drugs: one size fits all? Hospital Pharmacy Europe 2012 [cited 2020 Nov 19]; 62:59–63. Available from: URL: https://hospitalpharmacyeurope.com/news/editors-pick/market-access-of-orphan-drugs-one-size-fits-all/.

118. Largent EA. The many vs. the few - reply. Hastings Cent Rep 2012; 42(5):8-9.

119. Bonanno PV, Bucsics A, Simoens S, Martin AP, Oortwijn W, Gulbinovic J et al. Proposal for a regulation on health technology assessment in Europe - opinions of policy makers, payers and academics from the field of HTA. EXPERT REVIEW OF PHARMACOECONOMICS & OUTCOMES RESEARCH 2019; 19(3):251–61.

120. Cohen JP, Felix A. Are payers treating orphan drugs differently? J Mark Access Health Policy 2014; 2.

121. Douglas C, Wilcox E, Burgess M, Lynd LD. Why orphan drug coverage reimbursement decision-making needs patient and public involvement. HEALTH POLICY 2015; 119(5):588–96.

122. Hyry HI, Roos JCP, Cox TM. Orphan drugs: expensive yet necessary. QJM 2015; 108(4):269–72.

123. Rosenberg-Yunger ZRS, Daar AS, Thorsteinsdottir H, Martin DK. Priority setting for orphan drugs: An international comparison. HEALTH POLICY 2011; 100(1):25–34.

124. Angelis A, Kanavos P. Multiple Criteria Decision Analysis (MCDA) for evaluating new medicines in Health Technology Assessment and beyond: The Advance Value Framework. Social Science and Medicine 2017; 188:137–56.

125. Barak A, Shankar Nandi J. Orphan drugs: pricing, reimbursement and patient access. Intl J of Pharm & Health Mrkt 2011; 5(4):299–317.

126. Norheim OF, Baltussen R, Johri M, Chisholm D, Nord E, Brock D et al. Guidance on priority setting in health care (GPS-Health): the inclusion of equity criteria not captured by cost-effectiveness analysis. COST EFFECT RESOURCE ALLOCATION 2014; 12:18.

127. Annemans L, Aymé S, Yann Le Cam, Facey K, Gunther P, Nicod E et al. Recommendations from the European Working Group for Value Assessment and Funding Processes in Rare Diseases (ORPH-VAL). ORPHANET J RARE DIS 2017; 12:1–15. Available from: URL: http://search.ebscohost.com/login.aspx?direct=true&db=cin20&AN=121816714&site=ehost-live.

128. Degtiar I. A review of international coverage and pricing strategies for personalized medicine and orphan drugs. HEALTH POLICY 2017; 121(12):1240–8. Available from: URL: http://search.ebscohost.com/login.aspx?direct=true&db=cin20&AN=126312089&site=ehost-live.

129. Dintsios CM, Gerber A. Some essential clarifications: IQWiG comments on two critiques of the efficiency frontier approach. Health Economics (United Kingdom) 2010; 19(10):1139–41. Available from: URL: http://www.embase.com/search/results?subaction=viewrecord&from=export&id=L359700923.

130. He J, Song P, Kang Q, Zhang X, Hu J, Yang Y et al. Overview on social security system of rare diseases in China. BIOSCIENCE TRENDS 2019; 13(4):314–23.

131. Loblova O, Csanadi M, Ozieranski P, Kalo Z, King L, McKee M. Patterns of alternative access: Unpacking the Slovak extraordinary drug reimbursement regime 2012-2016. HEALTH POLICY 2019; 123(8):713–20.

132. Simoens S. Pricing and reimbursement of orphan drugs: the need for more transparency. ORPHANET JOURNAL OF RARE DISEASES 2011; 6.

133. Schwalm A, Danner M, Seidl A, Volz F, Dintsios CM, Gerber A. Wo steht die Kosten-Nutzen-Bewertung des IQWiG : Abgleich mit einem internationalen Referenzszenario? Bundesgesundheitsblatt Gesundheitsforschung Gesundheitsschutz 2010; 53(6):615–22.

134. Simoens S. How to assess the value of medicines? FRONTIERS IN PHARMACOLOGY 2010; 1:115.

135. Tony M, Wagner M, Khoury H, Rindress D, Papastavros T, Oh P et al. Bridging health technology assessment (HTA) with multicriteria decision analyses (MCDA): field testing of the EVIDEM framework for coverage decisions by a public payer in Canada. BMC HEALTH SERVICES RESEARCH 2011; 11:329.

136. Al-Attar M. TRAPPED - an insight into two sisters' struggle to access treatment for a rare genetic disease. ORPHANET JOURNAL OF RARE DISEASES 2018; 13(1):37.

137. Boon W, Moors EHM, Kuhlmann S, Smits, Ruud E. H. M. Demand articulation in intermediary organisations: The case of orphan drugs in the Netherlands. TECHNOLOGICAL FORECASTING AND SOCIAL CHANGE 2008; 75(5):644–71.

138. Bourke SM, Plumpton CO, Hughes D. Societal Preferences for Funding Orphan Drugs in the United Kingdom: An Application of Person Trade-Off and Discrete Choice Experiment Methods. Value Health 2018; 21(5):538–46.

139. Chowdhury MZI, Chowdhury MA. Canadian Health Care System: Who Should Pay for All Medically Beneficial Treatments? A Burning Issue. INT J HEALTH SERV 2018; 48(2):289–301. Available from: URL: http://search.ebscohost.com/login.aspx?direct=true&db=cin20&AN=129080413&site=ehost-live.

140. Doux J. Editorial: Barriers and Opportunities: A View across the Developmental Divide. Journal of Investigative Dermatology 2015; 135(9):2143–4. Available from: URL: http://www.embase.com/search/results?subaction=viewrecord&from=export&id=L605657879.

141. Forestier-Zhang L., Watts L., Turner A., Teare H., Kaye J., Barrett J. et al. Health-related quality of life and a cost-utility simulation of adults in the UK with osteogenesis imperfecta, X-linked hypophosphatemia and fibrous dysplasia. ORPHANET JOURNAL OF RARE DISEASES 2016; 11(1):1–9. Available from: URL: http://www.embase.com/search/results?subaction=viewrecord&from=export&id=L613418861.

142. Gammie T, Lu CY, Babar Z-U-D. Access to Orphan Drugs: A Comprehensive Review of Legislations, Regulations and Policies in 35 Countries. PLOS ONE 2015; 10(10):e0140002.

143. Gong S, Jin S. Current progress in the management of rare diseases and orphan drugs in China. INTRACTABLE & RARE DISEASES RESEARCH 2012; 1(2):45–52.

144. Guan X.-D., Zhang J., Man C., Ni B., Shi L.-W. How Far Have We Come? Challenges to Orphan Drug Access in China, 2011-2017. Journal of Pharmaceutical Sciences 2019; 108(6):2199–205. Available from: URL: http://www.embase.com/search/results?subaction=viewrecord&from=export&id=L2001554272.

145. Hyry HI, Stern AD, Cox TM, Roos JCP. Limits on use of health economic assessments for rare diseases. QJM-AN INTERNATIONAL JOURNAL OF MEDICINE 2014; 107(3):241–5.

146. Iskrov G, Raycheva RD, Stefanov R. Insight into reimbursement decision-making criteria in Bulgaria: implications for orphan drugs. Folia Med (Plovdiv) 2013; 55(3-4):80–6.

147. Kanters TA, Hakkaart L, Rutten-van Moelken, Maureen P. M. H., Redekop WK. Access to orphan drugs in western Europe: can more systematic policymaking really help to avoid different decisions about the same drug? EXPERT REVIEW OF PHARMACOECONOMICS & OUTCOMES RESEARCH 2015; 15(4):557–9.

148. McCabe C, Stafinski T, Menon D. Is it time to revisit orphan drug policies? BMJ 2010; 341:c4777.

149. Menon D, Stafinski T. Ultra-orphan drugs: can we afford the price. EXPERT OPINION ON ORPHAN DRUGS 2017; 5(8):611–2. Available from: URL: http://www.embase.com/search/results?subaction=viewrecord&from=export&id=L616897862.

150. Morel T, Simoens S. Coverage of Orphan Drugs. In: Ethgen O, Staginnus U, editors. FUTURE OF HEALTH ECONOMICS. 2 PARK SQ, MILTON PARK, ABINGDON OX14 4RN, OXFORD, ENGLAND: ROUTLEDGE; 2017. p. 109–21.

151. Mrsić M., Nola M. Rare diseases in Croatia - Lesson learned from Anderson-Fabry disease. CROATIAN MEDICAL JOURNAL 2008; 49(5):579–81. Available from: URL: http://www.embase.com/search/results?subaction=viewrecord&from=export&id=L352773868.

152. Oral M, Ozcelikay G. Ethical Overview of Pharmaceutical Industry Policies in Turkey from Various Perspectives. TURKISH JOURNAL OF PHARMACEUTICAL SCIENCES 2017; 14(3):264–73.

153. Picavet E, Cassiman D, Simoens S. Reimbursement of orphan drugs in Belgium: what (else) matters? ORPHANET JOURNAL OF RARE DISEASES 2014; 9:139.

154. Ramalle-Gomara E, Ruiz E, Quinones C, Andres S, Iruzubieta J, Gil-de-Gomez J. General knowledge and opinion of future health care and non-health care professionals on rare diseases. JOURNAL OF EVALUATION IN CLINICAL PRACTICE 2015; 21(2):198–201.

155. Rhee TG. Policymaking for Orphan Drugs and Its Challenges. AMA J Ethics 2015; 17(8):776–9.

156. Sandman L, Hofmann B. Why We Don’t Need “Unmet Needs”! On the Concepts of Unmet Need and Severity in Health-Care Priority Setting. Health Care Analysis 2019; 27(1):26–44.

157. Tilles S.A., Borish L. Author's response. Annals of Allergy, Asthma and Immunology 2012; 109(2):151. Available from: URL: http://www.embase.com/search/results?subaction=viewrecord&from=export&id=L365337411.

158. Torrent-Farnell J, Comellas M, Poveda JL, Abaitua I, Gutierrez-Solana LG, Perez-Lopez J et al. The view of experts on initiatives to be undertaken to promote equity in the access to orphan drugs and specialised care for rare diseases in Spain: A Delphi consensus. HEALTH POLICY 2018; 122(6):590–8.

159. Wells RJ. The many vs. the few. Hastings Cent Rep 2012; 42(5):7; author reply 8-9.

160. Wong-Rieger D, Rieger F. Health Policies for Orphan Diseases: International Comparison of Regulatory, Reimbursement and Health Services Policies. In: Bali RK, Bos L, Gibbons MC, Ibell, SR, editors. RARE DISEASES

IN THE AGE OF HEALTH 2.0. HEIDELBERGER PLATZ 3, D-14197 BERLIN, GERMANY: SPRINGER-VERLAG BERLIN; 2014. p. 267–77 (Communications in Medical and Care Compunetics).

161. Breaking the Access Deadlock to Leave No One Behind; January 2018 [cited 6.5.20]. Available from: URL: http://download2.eurordis.org.s3.amazonaws.com/positionpapers/eurordis_access_position_paper_final_4122017.pdf.

162. Transparency and Health Technology Assessment cooperation as proposed by the Regulation are the only real antidote to secrecy and political games; March 2018 [cited 6.5.20]. Available from: URL: http://download2.eurordis.org.s3.amazonaws.com/positionpapers/Statement_final.pdf.

163. GKV Spitzenverband. Nutzen und Schaden auch bei Arzneimitteln gegen seltene Krankheiten vollständig prüfen. Berlin; 2016 [cited 5.5.20]. Available from: URL: https://www.gkv-spitzenverband.de/presse/pressemitteilungen_und_statements/pressemitteilung_339584.jsp.

164. Annemans L, Cleemput I, Hulstaert F, Simoens S. Valorising and creating access to innovative medicines in the European union. FRONTIERS IN PHARMACOLOGY 2011; 2:57.

165. van Weely S, Leufkens HG. Priority Medicines for Europe and the World"A Public Health Approach to Innovation": Update on 2004 Background Paper; 2013 Mar 12 [cited 2020 Nov 20]. Available from: URL: https://www.who.int/medicines/areas/priority_medicines/BP6_19Rare.pdf.

166. Windeler J, Lange S. Nutzenbewertung in besonderen Situationen--Seltene Erkrankungen. Z Evid Fortbild Qual Gesundhwes 2008; 102(1):25–30.

167. Connolly M.P., Panda S., Patris J., Hazenberg B.P.C. Estimating the fiscal impact of rare diseases using a public economic framework: A case study applied to hereditary transthyretin-mediated (hATTR) amyloidosis. ORPHANET JOURNAL OF RARE DISEASES 2019; 14(1). Available from: URL: http://www.embase.com/search/results?subaction=viewrecord&from=export&id=L629346438.

168. Gross ML. Ethics, policy, and rare genetic disorders: the case of Gaucher disease in Israel. THEORETICAL MEDICINE AND BIOETHICS 2002; 23(2):151–70.

169. Whitty JA, Littlejohns P. Social values and health priority setting in Australia: an analysis applied to the context of health technology assessment. Health Policy 2015; 119(2):127–36.

170. Stafinski T, Menon D, Davis C, McCabe C. Role of centralized review processes for making reimbursement decisions on new health technologies in Europe. Clinicoecon Outcomes Res 2011; 3:117–86.

171. de Solà Morales O. Funding orphan medicinal products beyond price: sustaining an ecosystem. EUROPEAN JOURNAL OF HEALTH ECONOMICS 2019; 20(9):1283–6. Available from: URL: http://www.embase.com/search/results?subaction=viewrecord&from=export&id=L627200718.

172. Chim L, Salkeld G, Kelly P, Lipworth W, Hughes D, Stockler MR. Community views on factors affecting medicines resource allocation: cross-sectional survey of 3080 adults in Australia. AUST HEALTH REV 2019;

43(3):254–60. Available from: URL:
http://search.ebscohost.com/login.aspx?direct=true&db=cin20&AN=136807097&site=ehost-live.

173. Denis A, Mergaert L, Fostier C, Cleemput I, Simoens S. Issues surrounding orphan disease and orphan drug policies in Europe. APPLIED HEALTH ECONOMICS AND HEALTH POLICY 2010; 8(5):343–50.

174. Denis A, Mergaert L, Fostier C, Cleemput I., Hulstaert F, Simoens S. Critical assessment of Belgian reimbursement dossiers of orphan drugs. PHARMACOECONOMICS 2011; 29(10):883–93. Available from: URL: http://www.embase.com/search/results?subaction=viewrecord&from=export&id=L362542773.

175. Denis A, Mergaert L, Fostier C, Cleemput I, Simoens S. A comparative study of European rare disease and orphan drug markets. HEALTH POLICY 2010; 97(2-3):173–9.

176. Kanters TA, Van Der Ploeg A.T., Kruijshaar M.E., Rizopoulos D., Redekop WK, Rutten-van Moelken, Maureen P. M. H. et al. Cost-effectiveness of enzyme replacement therapy with alglucosidase alfa in adult patients with Pompe disease. ORPHANET JOURNAL OF RARE DISEASES 2017; 12(1). Available from: URL: http://www.embase.com/search/results?subaction=viewrecord&from=export&id=L619682106.

177. Lasalvia P, Prieto-Pinto L, Moreno M, Castrillon J, Romano G, Garzon-Orjuela N et al. International experiences in multicriteria decision analysis (MCDA) for evaluating orphan drugs: a scoping review. EXPERT REVIEW OF PHARMACOECONOMICS & OUTCOMES RESEARCH 2019; 19(4):409–20.

178. Li X.-Q., Peng X.-X., Gong C.-X. Access to Orphan Drugs is a Challenge for Sustainable Management of Cystinosis in China. CHINESE MEDICAL JOURNAL 2018; 131(19):2388–9. Available from: URL: http://www.embase.com/search/results?subaction=viewrecord&from=export&id=L624093740.

179. Linley WG, Hughes D. Societal views on NICE, cancer drugs fund and value-based pricing criteria for prioritising medicines: a cross-sectional survey of 4118 adults in Great Britain. HEALTH ECONOMICS 2013; 22(8):948–64.

180. Sarnola K, Ahonen R, Martikainen JE, Timonen J. Policies and availability of orphan medicines in outpatient care in 24 European countries. EUROPEAN JOURNAL OF CLINICAL PHARMACOLOGY 2018; 74(7):895–902.

181. Towse A, Barnsley P. Approaches to identifying, measuring, and aggregating elements of value. INTERNATIONAL JOURNAL OF TECHNOLOGY ASSESSMENT IN HEALTH CARE 2013; 29(4):360–4. Available from: URL: http://www.embase.com/search/results?subaction=viewrecord&from=export&id=L370412690.

182. Wagner M, Samaha D, Casciano R, Brougham M, Abrishami P, Petrie C et al. Moving Towards Accountability for Reasonableness - A Systematic Exploration of the Features of Legitimate Healthcare Coverage Decision-Making Processes Using Rare Diseases and Regenerative Therapies as a Case Study. INTERNATIONAL JOURNAL OF HEALTH POLICY AND MANAGEMENT 2019; 8(7):424–43.

183. Min R, Zhang X, Fang P, Wang B, Wang H. Health service security of patients with 8 certain rare diseases: evidence from China's national system for health service utilization of patients with healthcare insurance. ORPHANET JOURNAL OF RARE DISEASES 2019; 14(1):204.

184. White W. A rare disease patient/caregiver perspective on fair pricing and access to gene-based therapies. GENE THERAPY 2019.

185. Prevot J, Watters D. HTA's and access to rare diseases therapies: The view from the PID community. Pharmaceuticals, Policy and Law 2011; 13(3,4):177–81.

186. Magalhaes M. Can Severity Outweigh Smaller Numbers? A Deliberative Perspective from Canada. VALUE IN HEALTH 2018; 21(5):532–7.

187. Chim L, Salkeld G, Kelly P, Lipworth W, Hughes D, Stockler MR. Societal perspective on access to publicly subsidised medicines: A cross sectional survey of 3080 adults in Australia. PLOS ONE 2017; 12(3):e0172971.

188. Hughes-Wilson W, Palma A, Schuurman A, Simoens S. Paying for the Orphan Drug System: break or bend? Is it time for a new evaluation system for payers in Europe to take account of new rare disease treatments? ORPHANET JOURNAL OF RARE DISEASES 2012; 7.

189. Jena A, Lakdawalla D. Value Frameworks For Rare Diseases: Should They Be Different?; 2017 Apr 12 [cited 2020 Nov 17]. Available from: URL: https://www.healthaffairs.org/do/10.1377/hblog20170412.059563/full/.

190. Medić B., Divac N., Stopić B., Vujović K.S., Glišić A., Cerovac N. et al. The attitudes of medical students towards rare diseases: A cross-sectional study. Vojnosanitetski Pregled 2016; 73(8):703–13. Available from: URL: http://www.embase.com/search/results?subaction=viewrecord&from=export&id=L611514423.

191. Nicod E, Annemans L, Bucsics A, Lee A., Upadhyaya S, Facey K. HTA programme response to the challenges of dealing with orphan medicinal products: Process evaluation in selected European countries. HEALTH POLICY 2019; 123(2):140–51. Available from: URL: http://www.embase.com/search/results?subaction=viewrecord&from=export&id=L615296658.

192. Wagner M, Khoury H, Bennetts L, Berto P, Ehreth J, Badia X et al. Appraising the holistic value of Lenvatinib for radio-iodine refractory differentiated thyroid cancer: A multi-country study applying pragmatic MCDA. BMC Cancer 2017; 17(1):272.

193. Young A, Menon D, Street J, Al-Hertani W, Stafinski T. A checklist for managed access programmes for reimbursement co-designed by Canadian patients and caregivers. Health Expect 2018; 21(6):973–80.

194. Lowin J, Bergman A, Ray Chaudhuri K, Findley LJ, Roeder C, Schifflers M et al. A cost-effectiveness analysis of levodopa/carbidopa intestinal gel compared to standard care in late stage Parkinson's disease in the UK. J MED ECON 2011; 14(5):584–93. Available from: URL: http://search.ebscohost.com/login.aspx?direct=true&db=cin20&AN=108196055&site=ehost-live.

195. Malinowski KP, Kawalec PL, Trabka W, Sowada C, Pilc A. Reimbursement of Orphan Drugs in Europe in Relation to the Type of Authorization by the European Medicines Agency and the Decision Making Based on Health Technology Assessment. FRONTIERS IN PHARMACOLOGY 2018; 9.

196. Nicod E, Kanavos P. Scientific and Social Value Judgments for Orphan Drugs in Health Technology Assessment. INTERNATIONAL JOURNAL OF TECHNOLOGY ASSESSMENT IN HEALTH CARE 2016; 32(4):218–32. Available from: URL: http://www.embase.com/search/results?subaction=viewrecord&from=export&id=L612179165.

197. The Lancet Neurology. Treating rare disorders: time to act on unfair prices. The Lancet Neurology 2017; 16(10):761. Available from: URL: http://www.embase.com/search/results?subaction=viewrecord&from=export&id=L618259662.

198. Winquist E, Bell CM, Clarke JTR, Evans G, Martin J, Sabharwal M et al. An evaluation framework for funding drugs for rare diseases. Value Health 2012; 15(6):982–6.

199. Hughes D. Orphan drugs revisited: Author's response [4]. QJM 2006; 99(5):350–1. Available from: URL: http://www.embase.com/search/results?subaction=viewrecord&from=export&id=L43651030.

200. Sheldon T. Dutch doctors call for EU evaluation of cost effectiveness of high cost orphan drugs. BMJ BR MED J (CLIN RES ED) 2012; 345:e5461-e5461. Available from: URL: http://search.ebscohost.com/login.aspx?direct=true&db=cin20&AN=107821151&site=ehost-live.

201. The Lancet. Rare diseases need sustainable options. The Lancet 2020; 395(10225):660. Available from: URL: http://www.embase.com/search/results?subaction=viewrecord&from=export&id=L2005064868.

202. Vogler S, Paris V, Ferrario A, Wirtz VJ, Joncheere K de, Schneider P et al. How Can Pricing and Reimbursement Policies Improve Affordable Access to Medicines? Lessons Learned from European Countries. APPLIED HEALTH ECONOMICS AND HEALTH POLICY 2017; 15(3):307–21.

203. Teagarden J.R., Unger T.F., Hirsch G. Access and availability of orphan drugs in the United States: Advances or cruel hoaxes? EXPERT OPINION ON ORPHAN DRUGS 2014; 2(11):1147–50. Available from: URL: http://www.embase.com/search/results?subaction=viewrecord&from=export&id=L600418093.

204. Xin X.-X., Guan X.-D., Shi L.-W. Catastrophic expenditure and impoverishment of patients affected by 7 rare diseases in China. ORPHANET JOURNAL OF RARE DISEASES 2016; 11(1). Available from: URL: http://www.embase.com/search/results?subaction=viewrecord&from=export&id=L610858538.

205. Kesselheim AS, McGraw S, Thompson L, O'Keefe K, Gagne JJ. Development and use of new therapeutics for rare diseases: views from patients, caregivers, and advocates. PATIENT 2015; 8(1):75–84.

206. Farrugia A, O'Mahony B, Cassar J. Health technology assessment and haemophilia. HAEMOPHILIA 2012; 18(2):152–7.

207. Loewenstein G. The many vs. the few. Hastings Cent Rep 2012; 42(5):7-8; author reply 8-9.

208. Menzel PT. The many vs. the few. Hastings Cent Rep 2012; 42(5):5-6; author reply 8-9.

209. Allotey PA, Allotey-Reidpath CD, Reidpath DD. Health systems implications of rare genetic conditions in low- and middle-income countries: a case study approach. CRIT PUBLIC HEALTH 2018; 28(2):248–52. Available from: URL: http://search.ebscohost.com/login.aspx?direct=true&db=cin20&AN=127161649&site=ehost-live.

210. Caulfield T, Toews M. Rare Diseases and Resource Allocation Policy: The Role of Canadian Legal and Ethical Norms. U.B.C. L. Rev. 2016; 49:789. Available from: URL: https://heinonline.org/HOL/Page?public=true&handle=hein.journals/ubclr49&div=22&start_page=789&collection=journals&set_as_cursor=65&men_tab=srchresults.

211. Rachul C, Caulfield T. The media and access issues: content analysis of Canadian newspaper coverage of health policy decisions. ORPHANET JOURNAL OF RARE DISEASES 2015; 10.

212. Rachul C, Toews M, Caulfield T. Controversies with Kalydeco: Newspaper coverage in Canada and the United States of the cystic fibrosis “wonder drug”. JOURNAL OF CYSTIC FIBROSIS 2016; 15(5):624–9.

213. Balfour-Lynn I.M. Personalised medicine in cystic fibrosis is unaffordable. Paediatric Respiratory Reviews 2014; 15(S1):2–5. Available from: URL: http://www.embase.com/search/results?subaction=viewrecord&from=export&id=L53131881.

214. Bavisetty S., Grody W.W., Yazdani S. Emergence of pediatric rare diseases: Review of present policies and opportunities for improvement. RARE DISEASES 2013; 1. Available from: URL: http://www.embase.com/search/results?subaction=viewrecord&from=export&id=L372086588.

215. Das AM, Lagler F, Beck M, Scarpa M, Lampe C. Lysosomal Storage Diseases: Challenges in Multiprofessional Patient Care with Enzyme Replacement Therapy. KLINISCHE PADIATRIE 2017; 229(3):168–74.

216. Saint-Raymond A, Llinares J. Orphan medicines: a success with a challenging future. EXPERT OPINION ON ORPHAN DRUGS 2013; 1(3):185–7.

217. Zallen DT. The many vs. the few. Hastings Cent Rep 2012; 42(5):4-5; author reply 8-9.

218. Boon W, Martins L, Koopmanschap M. Governance of conditional reimbursement practices in The Netherlands. Health Policy 2015; 119(2):180–5.

219. Schlander M. The use of cost-effectiveness by the National Institute for Health and Clinical Excellence (NICE): no(t yet an) exemplar of a deliberative process. Journal of Medical Ethics 2008; 34(7):534–9.

220. Cavalier GM. Pushing Parentless Pharmaceuticals: Toward an International Home for Orphan Drugs and a Cure for Zebra Diseases. Law & Pol'y Int'l Bus. 1995; 27:447. Available from: URL: https://heinonline.org/HOL/Page?public=true&handle=hein.journals/geojintl27&div=19&start_page=447&collection=journals&set_as_cursor=19&men_tab=srchresults.

221. Perehudoff K, Toebes B, Hogerzeil H. A human rights-based approach to the reimbursement of expensive medicines. BULLETIN OF THE WORLD HEALTH ORGANIZATION 2016; 94(12):935.

222. Stafinski T, Menon D, Philippon DJ, McCabe C. Health technology funding decision-making processes around the world: the same, yet different. PHARMACOECONOMICS 2011; 29(6):475–95.

223. Taylor DW. Redressing the inequities in canadian pharmacare. Healthcare Management Forum 2015; 28(2):50–4. Available from: URL: http://www.embase.com/search/results?subaction=viewrecord&from=export&id=L617660644.

224. IPA position paper: Patient Access to Approved Therapies [cited 6.5.20]. Available from: URL: https://www.worldpompe.org/images/pdfs/ipa%20position%20paper%20-%20patient%20access.pdf.

225. Special Eurobarometer 361: European awareness of rare diseases; 2011 [cited 2020 Nov 16]. Available from: URL: https://ec.europa.eu/health//sites/health/files/rare_diseases/docs/ebs_361_en.pdf.

226. Norheim OF. Ethical priority setting for universal health coverage: challenges in deciding upon fair distribution of health services. BMC MEDICINE 2016; 14.

227. Bourdoncle M, Juillard-Condat B, Taboulet F. Patient access to orphan drugs in France. ORPHANET JOURNAL OF RARE DISEASES 2019; 14(1):47.

228. McCabe C. The many vs. the few. Hastings Cent Rep 2012; 42(5):6-7; author reply 8-9.

229. Whitty JA, Lancsar E, Rixon K, Golenko X, Ratcliffe J. A systematic review of stated preference studies reporting public preferences for healthcare priority setting. PATIENT 2014; 7(4):365–86.

230. Jessop E, Upadhyaya S. Ultra orphan drugs: the NHS model for managing extremely rare diseases. EXPERT OPINION ON ORPHAN DRUGS 2014; 2(12):1301–8.

231. Kolasa K, Zwolinski KM, Kalo Z, Hermanowski T. Potential impact of the implementation of multiple-criteria decision analysis (MCDA) on the Polish pricing and reimbursement process of orphan drugs. ORPHANET JOURNAL OF RARE DISEASES 2016; 11.

232. Lelgemann M, Francke R. Rare diseases in professional health care. Bundesgesundheitsblatt Gesundheitsforschung Gesundheitsschutz 2008; 51(5):509–18.

233. Sassi F, Archard L, Le Grand J. Equity and the economic evaluation of healthcare. Health Technology Assessment 2001; 5(3):1–138.

234. Lüthi U. Solidarity is what is needed. Krankenpfl Soins Infirm 2015; 108(5):1.

235. Polisena J, Burgess M, Mitton C, Lynd LD. Engaging the Canadian public on reimbursement decision-making for drugs for rare diseases: a national online survey. BMC HEALTH SERVICES RESEARCH 2017; 17.

236. Schuller Y., Hollak C.E.M., Biegstraaten M. The quality of economic evaluations of ultra-orphan drugs in Europe - A systematic review. ORPHANET JOURNAL OF RARE DISEASES 2015; 10(1). Available from: URL: http://www.embase.com/search/results?subaction=viewrecord&from=export&id=L612265876.

237. Winquist E, Coyle D, Clarke JTR, Evans G, Seager C, Chan W et al. Application of a Policy Framework for the Public Funding of Drugs for Rare Diseases. JOURNAL OF GENERAL INTERNAL MEDICINE 2014; 29(3):S774-S779.

238. Drummond M, Tarricone R, Torbica A. Assessing the added value of health technologies: reconciling different perspectives. Value Health 2013; 16(1 Suppl):S7-13. Available from: URL: http://www.sciencedirect.com/science/article/pii/S1098301512041587.

239. Lumry WR. Hereditary Angioedema: The Economics of Treatment of an Orphan Disease. FRONTIERS IN MEDICINE 2018; 5.

240. Salzman R, Cook F, Hunt T, Malech HL, Reilly P, Foss-Campbell B et al. Addressing the Value of Gene Therapy and Enhancing Patient Access to Transformative Treatments. Molecular Therapy 2018; 26(12):2717–26.

241. Moors EHM, Faber J. Orphan drugs: Unmet societal need for non-profitable privately supplied new products. RESEARCH POLICY 2007; 36(3):336–54.

242. Herder M. When everyone is an orphan: against adopting a U.S.-styled orphan drug policy in Canada. Account Res 2013; 20(4):227–69.

243. Luzzatto L., Hyry HI, Schieppati A., Costa E., Simoens S, Schaefer F. et al. Outrageous prices of orphan drugs: a call for collaboration. The Lancet 2018; 392(10149):791–4. Available from: URL: http://www.embase.com/search/results?subaction=viewrecord&from=export&id=L2001272164.
